# The effects of housing density on mouse thermal physiology depend on sex and ambient temperature

**DOI:** 10.1016/j.molmet.2021.101332

**Published:** 2021-09-01

**Authors:** Vojtěch Škop, Cuiying Xiao, Naili Liu, Oksana Gavrilova, Marc L. Reitman

**Affiliations:** 1 Diabetes, Endocrinology, and Obesity Branch, National Institute of Diabetes and Digestive and Kidney Diseases, National Institutes of Health, Bethesda, MD 20892, USA; 2 Mouse Metabolism Core, National Institute of Diabetes and Digestive and Kidney Diseases, National Institutes of Health, Bethesda, MD 20892, USA

**Keywords:** Body temperature, Energy expenditure, Housing density, Sex differences, Bombesin receptor subtype-3 (BRS3, BB3), Ambient temperature, BRS3, bombesin receptor subtype-3, RER, respiratory exchange ratio, Ta, ambient temperature(s), Tb, core body temperature, TEE, total energy expenditure, TNP_D_, dark phase thermoneutral point, TNP_L_, light phase thermoneutral point

## Abstract

**Objective:**

To improve understanding of mouse energy homeostasis and its applicability to humans, we quantitated the effects of housing density on mouse thermal physiology in both sexes.

**Methods:**

Littermate wild type and *Brs3*-null mice were single- or group- (three per cage) housed and studied by indirect calorimetry with continuous measurement of core body temperature, energy expenditure, physical activity, and food intake.

**Results:**

At 23 °C, below thermoneutrality, single-housed males had a lower body temperature and unchanged metabolic rate compared to group-housed controls. In contrast, single-housed females maintained a similar body temperature to group-housed controls by increasing their metabolic rate. With decreasing ambient temperature below 27 °C, only group-housed mice decreased their heat conductance, likely due to huddling, thus interfering with the energy expenditure vs ambient temperature relationship described by Scholander. In a hot environment (35 °C), the single-housed mice were less heat stressed. Upon fasting, single-housed mice had larger reductions in body temperature, with male mice having more torpor episodes of similar duration and female mice having a similar number of torpor episodes that lasted longer. Qualitatively, the effects of housing density on thermal physiology of *Brs3*-null mice generally mimicked the effects in controls.

**Conclusions:**

Single housing is more sensitive than group housing for detecting thermal physiology phenotypes. Single housing increases heat loss and amplifies the effects of fasting or a cold environment. Male and female mice utilize different thermoregulatory strategies to respond to single housing.

## Highlights

• Changing housing density changes thermal physiology and metabolic rate.
• Singly housed mice are more affected by fasting and by cold temperatures.
• Single housing is more sensitive than group housing for detecting thermal phenotypes.
• Certain principles of thermal physiology are masked by group housing.
• Male and female mice respond differently to single housing.

## Graphical abstract

**Figure:**
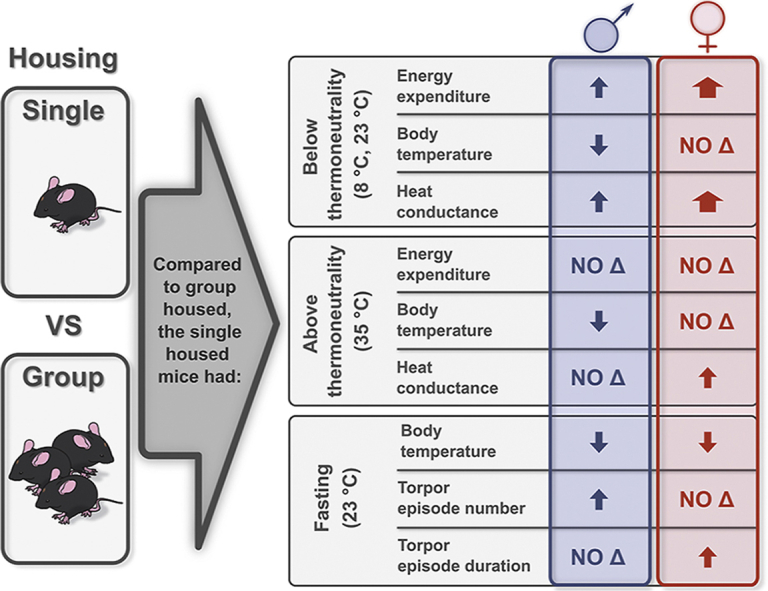

## 1 Introduction

Mice are a widely used research model for human diseases, including obesity and diabetes. Mice are genetically tractable and easy to study, and genes and metabolic pathways are very highly conserved between the species. On the other hand, the ∼3000-fold difference in body weight causes huge differences in thermal physiology and energy homeostasis [1,2]. For example, in mice, the mass-specific metabolic rate is ∼7-fold higher and the surface area:volume ratio is ∼14-fold higher than in humans. Thus better understanding of mouse thermal physiology is needed to drive experimental design, guide interpretation of results, and generally improve applicability of mouse observations to humans. Recent efforts to make mice a better model have focused on housing temperature [[3], [4], [5], [6]]. Another environmental factor that impacts thermal physiology is housing density: group vs single housing.

Vivarium conditions that do not allow natural behaviors can cause stress and impact experimental results [7,8]. In a natural environment, a territory is occupied by one dominant male mouse, several females, and their sexually non-mature offspring [9]. Reproducing this environment in the laboratory is typically not feasible, leaving two imperfect choices. One is single-sex group housing, which allows for some social interaction, but aggression and fighting often occur in male groups. Another choice, single housing, is currently essential for measuring the food intake or energy expenditures of individual mice, but is considered stressful since it does not permit social interaction.

Under cold conditions, single-housed mice have increased metabolic rates and food intake [10,11], as well as more active brown adipose tissue (BAT) with increased uncoupling protein 1 (UCP1) levels [11,12] compared to group-housed controls. There is little information on the effect of group housing on core body temperature (Tb); it is not addressed in standard references [[13], [14], [15], [16], [17]]. In one study, more mice per cage increased cage ambient temperature (Ta) but did not affect Tb of female mice [18], and in a temperature preference test, group-housed females selected a slightly lower Ta than single-housed controls [19]. The availability of telemetry systems that allow Tb monitoring of multiple mice per cage prompted us to investigate how housing density affects thermal physiology, including Tb.

Here, we compared the effects of housing density using C57BL/6J mice, one of the most-commonly studied strains in metabolic research. Both male and female mice were studied, since females have a higher Tb and regulate Tb differently than do males [20,21]. We also analyzed mice lacking bombesin receptor subtype-3 (BRS3, bombesin-like receptor 3, BB3), an orphan G protein-coupled receptor that regulates energy homeostasis, which includes metabolic rate, Tb, food intake, and body weight [[22], [23], [24], [25], [26]]. We find clear sex-dimorphic differences in thermal biology between single and group housing.

## 2 Materials and methods

### 2.1 Animals

Mice were housed (including during indirect calorimetry) in Tecniplast 1284 cages with a 12:12-h dark:light cycle (lights on at 0600) in a clean, conventional facility with ∼95 g of wood-chip bedding (7090 Teklad sani-chips, Envigo, Indianapolis, IN), without enrichment or nesting materials, and with water and chow (NIH-07 Envigo Inc, Madison, WI; 3.1 metabolizable kcal/g, food quotient 0.909) provided *ad libitum*. Housing was at 21–23 °C when not otherwise stated. Experiments were approved by the NIDDK Institutional Animal Care and Use Committee (protocol K016-DEOB-20). Mice were of a C57BL/6J background. The *Brs3*-null allele is *Brs3*^*tm2Rei*^/6J (JAX 032580) [27] and mice were littermate progeny of male *Brs3*^*-/y*^ × female *Brs3*^*+/−*^ matings, using *Brs3*^*+/−*^ mice as female controls.

Tb telemetry sensors were implanted intraperitoneally under isoflurane anesthesia (5% induction, 1.2% maintenance; Baxter Healthcare Corporation, Deerfield, IL) with Prevail (flunixin meglumine) analgesia (2.2 mg/kg sc at operation).

### 2.2 Experimental design

**Figure 1:**
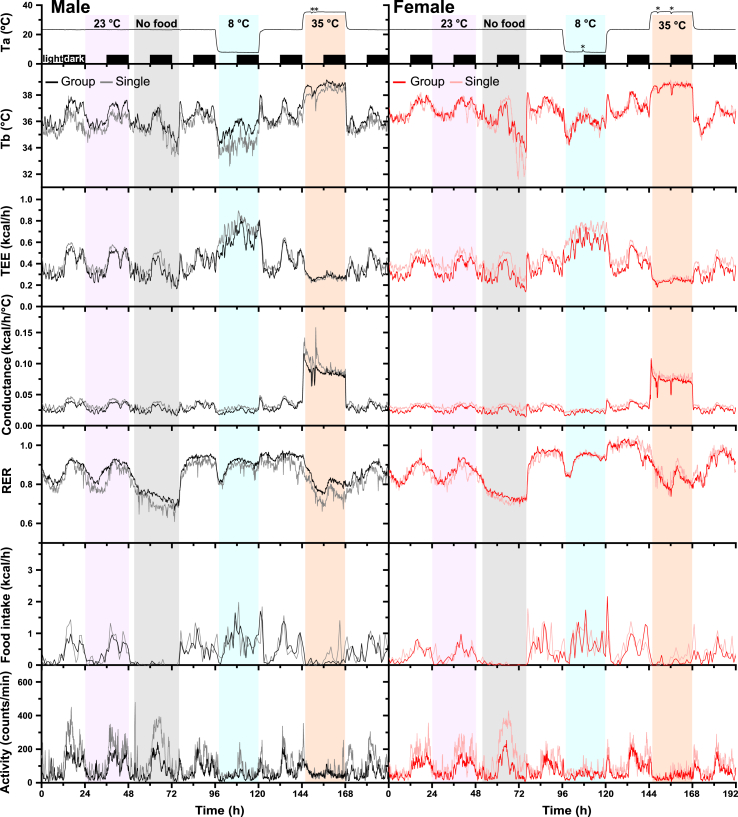
Effect of housing density on thermal physiology. Mice were studied in a home-cage indirect calorimetry system for eight days. Ambient temperature (Ta), body temperature (Tb), total energy expenditure (TEE), heat conductance, respiratory exchange ratio (RER), food intake, and physical activity were monitored continuously. Time 0 is 0600, onset of light phase, with light/dark phase indicated in the Ta panel. Analysis intervals are indicated: 23 °C (violet shading), 24-h fast (grey shading), 8 °C (blue shading), and 35 °C (red shading). Data are group means (n = 5 to 18 mice) using 10-min bins (Ta, Tb, TEE, conductance, RER, activity) or 1-h bins (food intake). Group housing density was three mice per cage. Cage handling to adjust water dispensers is indicated by ∗. For visual clarity, error bars are omitted.

Mice were randomized into six cages of each of the housing (one or three mice/cage) and genotype (wild type or *Brs3*-null) conditions, totaling 24 cages and 48 mice of each sex. Male mice were studied starting at 10 weeks of age and females starting at 15 weeks of age. The sequential design allowed reuse of the same set of TS100 telemetry sensors. The 48 mice were implanted with sensors over the course of three days and recovered for at least five days. Half were studied using indirect calorimetry for eight days (including fasting and cold and hot exposures), followed by another eight days of only Tb measurement at room temperature (including a 24-h fast) (Figure 1). The other half were studied in reverse order (Tb measurement only followed by indirect calorimetry) and the two half cohorts were pooled for analysis. The 12-cage capacity of the calorimetry system necessitated the crossover design.

In a separate experiment, group- (n = 18) and single- (n = 6) housed 16-week-old male and 18-week-old female C57BL/6J mice were exposed to a gradient Ta change in CLAMS-HC as previously described [6]. In this case, Tb was measured by G2 E-Mitter transponders. Since this system can only use one sensor per cage, just one of the three mice in each group cage was implanted.

At the end of the experiment (after at least four days at 23 °C), mice were anesthetized using ketamine/xylazine (100/10 mg/kg), blood was collected from the retro-orbital sinus, and mice were euthanized by cervical dislocation. BAT, inguinal white adipose tissue (iWAT), and gonadal WAT (gWAT) were collected. The following assay kits were used: leptin, R&D Systems MOB00B; T3, Calbiotech T3043T-100; and T4, Calbiotech T4044T-100. RNA from BAT and iWAT was quantitated using QuantStudio 7 Flex Real-Time PCR System (Applied Biosystems, Waltham, MA). *Ucp1* was normalized to *Tbp*. Primers are: *Ucp1,* x306 (5′-ACTGCCACACCTCCAGTCATT) and x307 (5′-CTTTGCCTCACTCAGGATTGG) as well as *Tbp,* x764 (5′-TTTGTGCCAGATACATTCCG) and x765 (5′-AACAATTTACAAGCTGCGTTT).

### 2.3 Characterization of telemetry sensors

Prior to implantation, we compared CubiSens TS100 (CubeWorks, Ann Arbor, MI) and G2 E-Mitter transponders (Starr Life Sciences, Oakmont, PA) Tb telemetry sensors. The absolute value of the difference in temperature reading between pairs of TS100 sensors was 0.047 ± 0.020 °C (mean ± SD, n = 6 pairs measured for three days) compared to 0.125 ± 0.080 °C (n = 4 pairs) for G2 E-Mitters. The within-pair SD of the difference was 0.019 ± 0.008 °C (mean ± SD) for TS100s and 0.080 ± 0.021 °C for G2 E-Mitters. Thus the TS100s exhibit less variability than the G2 E-Mitters.

The TS100 sensors were programmed to report Tb nominally every 4 min, although the exact interval varies by sensor. The measured intrinsic sampling interval was 4.14 ± 0.19 min (mean ± SD; n = 52; using 72 h of data), giving a between–sensor coefficient of variation of 4.70%, compared to within-sensor coefficients of variation of 1.80 ± 0.58% (mean ± SD). TS100 sensors with a mean measurement interval >15 min (typically due to battery depletion) were excluded from analysis. After these exclusions, the mean measurement intervals were 4.9 min in males and 6.6 min in females. Tb data were analyzed in 10 min bins (approximately twice the sampling interval), giving about two measurements/sensor/bin. For mice with two implanted intraperitoneal sensors, data from one sensor was randomly selected, except when specifically comparing the two sensors, with no change in the results if the other sensor was used instead.

We found that both TS100 and G2 E-Mitter transponders interfere with body composition measurement by EchoMRI (EchoMRI LLC, Houston, TX).

### 2.4 Characterization of body temperature telemetry

To better understand in vivo Tb measurements with TS100, two sensors were implanted in some mice. The absolute value of the difference between the two sensors within a mouse was 0.098 ± 0.141 °C (mean ± SD, n = 4 mice) and the within-mouse SD of the difference was 0.324 ± 0.048 °C (mean ± SD). The likely source of variation between the two intraperitoneal sensors in the same mouse is sensor position (see [28]). This is consistent with our observation that the difference between the two sensors sometimes shifted over time. The TS100s do not have an attachment point and were not sutured to the peritoneum. In our experience, suturing G2 E-Mitters did not reduce variability and the sensors are often not attached at necropsy weeks or months later.

Tb span is the difference between the Tb 95th and 5th percentiles within 24-h intervals [27]. We obtained some anomalously small Tb span measurements, present in the mice with the lowest 24-h mean Tb (Figure S1). The likely explanation is that a low 24-h mean Tb identifies sensors positioned in a way that includes a Ta contribution, which reduces the measured Tb span.

### 2.5 Indirect calorimetry

Total energy expenditure (TEE), respiratory exchange ratio (RER), physical activity (infrared beam break, 1 inch spacing; each count is the “total” beam breaks summing the X and Y directions), and food (from a hanging feeder) and water intake were measured by a “home cage” Comprehensive Lab Animal Monitoring System (CLAMS-HC using Oxymax v5.52, Columbus Instruments, Columbus, OH). Ta and relative humidity inside each cage were monitored continuously. Calorimetry parameters are: 7.75 L cage volume, 0.9 L/min flow rate, 0.6 L/min sampling flow, 15 s settle time, 5 s measure time, with each chamber sampled every 260 s. For group-housed mice, TEE, physical activity, and food intake values were divided by the number of mice in the cage. For physical activity, this could be an underestimate in the case of non-detection of coincident activity of more than one mouse. The cages were located in a single environmental chamber set to 6, 22, and 34.5 °C to achieve temperatures inside the mouse cages of, nominally, 8, 23, and 35 °C respectively (see 3.1). Whole-body heat conductance was calculated as TEE/(Tb–Ta) [29], with the measured cage Ta used for heat conductance calculations.

VCO_2_ data for half of the females at Ta = 35 °C were lost due to failure of the CO_2_ sensor. Since the other mice for this condition were studied successfully, we used their measured RER of 0.842 ± 0.055 (SD) to calculate the TEE from the measured VO2. We note that the caloric content of carbohydrate (4.981 kcal/L O_2_ for RER = 1.00) is only slightly higher than that of fat (4.693 kcal/L O_2_ for RER = 0.71); thus each 0.01 unit increase in RER increases TEE by only 0.21% [30,31]. Even if the actual RER is slightly different from that assumed, the effect on TEE is trivial.

To examine the effects of adjusting TEE for body weight, we calculated the slope of the TEE vs body weight regression line [16]. In 30 of the 32 datasets (23 °C/35 °C, light/dark, group/single, male/female, control/*Brs3*-null), the slope was not significantly different from zero at P < 0.05 before any multiplicity correction. This is presumably due to the small effect of body weight on TEE over the narrow range of body weights studied. Thus no adjustment of TEE for body weight was made.

### 2.6 Statistical analyses

Data analysis was performed using R (version 4.0.2), SAS (version 9.4; SAS Institute, Cary, NC, USA), and Prism (version 8.1.0; GraphPad Software, Inc.). Effects of Ta on TEE and Tb were analyzed by segmented line regression as described [6]. Data are presented as mean ± SEM unless SD is indicated. Two-way analysis of variance (ANOVA) and post-hoc analysis with Šidák's multiple comparisons test were used to test statistical significance of housing (group vs single), genotype (control vs *Brs3*-null), and their interaction.

## 3 Results

### 3.1 Characteristics of single and group housing

We studied the effects of housing density by comparing three mice per cage (“group”) vs one mouse per cage (“single”) under various conditions, including a 24-h fast and cold (8 °C) and hot (35 °C) ambient temperatures (Ta) (Figure 1). Both housing densities were studied for four groups of littermate mice: male controls (WT), male *Brs3* nulls (*Brs3*^*-/y*^), female controls (*Brs*^*+/-*^), and female *Brs3* nulls (*Brs3*^*−/−*^). We first report the results of wild type (3.2–3.7) and then those of the *Brs3-*null (3.8).

The Ta inside the mouse cages was not majorly affected by housing density (Table 1). In contrast, the relative humidity in the group cages was significantly higher than in the single-mouse cages (Table 1).

**Table 1:** Measured cage ambient temperature (Ta) and relative humidity.

		Males							Females						
	Nominal	WT	WT	*Brs3*^*-/y*^	*Brs3*^*-/y*^	2-way ANOVA *P* value			*Brs3*^*+/−*^	*Brs3*^*+/−*^	*Brs3*^*−/−*^	*Brs3*^*−/−*^	2-way ANOVA *P* value		
	Ta	group	single	group	single	H	G	H × G	group	single	group	single	H	G	H × G
Measured Ta (°C)	8 °C	8.3 ± 0.2	8.3 ± 0.2	7.5 ± 0.6	7.6 ± 0.4	0.95	0.04	0.94	8.7 ± 0.2	7.9 ± 0.1	8.2 ± 0.3	7.4 ± 0.4	0.003	0.053	0.98
	23 °C	23.5 ± 0.1	23.5 ± 0.1	23.0 ± 0.4	23.2 ± 0.2	0.69	0.07	0.78	23.7 ± 0.1	23.4 ± 0.1	23.7 ± 0.2	23.0 ± 0.3	0.013	0.36	0.30
	35 °C	35.4 ± 0.1	35.4 ± 0.1	35.2 ± 0.2	35.3 ± 0.1	0.58	0.16	0.54	35.4 ± 0.1	35.4 ± 0.1	35.5 ± 0.1	35.2 ± 0.1	0.11	0.64	0.030
Relative humidity (%)	8 °C	97.7 ± 0.8	78.6 ± 0.8	95.6 ± 2.3	78.4 ± 1.3	<0.0001	0.41	0.48	97.5 ± 1.7	79.0 ± 0.7	89.2 ± 3.5	85.4 ± 1.3	<0.0001	0.64	0.003
	23 °C	83.7 ± 1.1	64.8 ± 1.5	82.4 ± 4.8	62.3 ± 2.6	<0.0001	0.48	0.83	87.2 ± 3.4	68.6 ± 4.3	80.4 ± 3.7	78.1 ± 4.7	0.022	0.74	0.064
	35 °C	87.3 ± 1.6	72.2 ± 1.6	91.3 ± 4.5	68.8 ± 2.9	<0.0001	0.92	0.20	85.7 ± 3.2	63.2 ± 1.8	79.7 ± 6.1	73.1 ± 0.8	0.001	0.60	0.045

For each cage, the continuously measured in-cage Ta and relative humidity were averaged over 24 h. P values from two-way ANOVA: H is housing, G is genotype, and H × G is the housing by genotype interaction. Data are mean ± SEM, n = 3–6/group.

Body weights of single- and group-housed mice were matched at sensor implantation; however, single-housed male (but not female) mice lost slightly more weight during recovery from surgery (Figure S2). Body weight was ∼2 g higher in male *Brs3-*null mice than controls, as expected [26]; female *Brs3-*null mice were also heavier at some time points.

**Figure 2:**
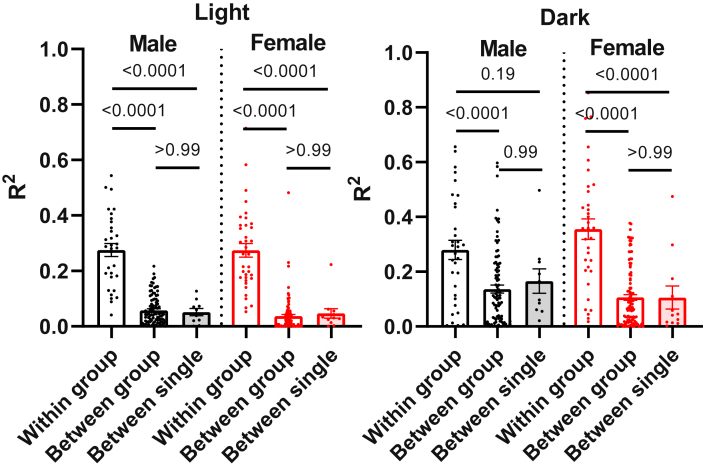
Correlation between body temperatures. At each time point, all possible pairwise Tb comparisons of mice in the same group cage (within group, n = 32–36), of mice in one group cage with mice in a different group cage (between group, n = 96–108), and of single-housed mice to other single-housed mice (between single, n = 10–12) were calculated separately for each sex/phase/genotype group. Initial analyses did not demonstrate a genotype effect, so results are reported by sex and phase, pooling genotypes. The R^2^ were calculated from three days of data (10-min bins, 173 ± 50 (SD) points) from mice housed at 23 °C. Data are mean ± SEM with one-way ANOVA adjusted P values as indicated.

To investigate the effect of group housing on interactions between mice, we used the correlation between the Tbs of pairs of mice. We compared mice sharing a group cage (“within group”) to two controls, mice in different group cages (“between group”) and mice in single cages (“between single”). The within-cage correlations were higher than the between-cage correlations by 6.4-fold in light and 2.7-fold in dark (Figure 2). Because minute-to-minute variation in Tb correlates with physical activity, these results indicate some synchronization of activity patterns among the mice in a group cage.

### 3.2 Effect of single housing at 23 °C on body temperature and energy expenditure

Tb is higher in the active (dark in mouse) phase compared to the inactive phase [32]. In our experiment, the dark–light difference was 0.78–1.38 °C in mice at 23 °C (Table S1). Thus the light and dark phases were analyzed separately.

Another property of Tb is that it is higher in females than in males [20]. This was generally true in both single and group housing (e.g., in the light phase, higher by ∼0.9 °C in single- and ∼0.5 °C in group-housed).

To quantitate short term (minute-to-minute) Tb variation, we calculated the largest Tb difference between any two mice within a group cage for each 10-min interval. At 23 °C this was 0.81 ± 0.26 °C (males, light), 0.98 ± 0.46 °C (males, dark), 0.74 ± 0.10 °C (females, light), and 0.84 ± 0.14 °C (females, dark) (mean ± SD, n = 12 cages, measured over 72 h), with no significant difference between sexes. This variation is about 3-fold greater than the variation observed between two sensors within the same mouse (which is likely due to sensor position) and an order of magnitude greater than the intrinsic TS100 variability (see 2.3 and 2.4). Physical activity is likely the major contributor to minute-to-minute Tb variation in group-housed mice, as it is in single-housed mice. To minimize effects of this short-term variation, we analyzed 12-h measurement intervals.

**Figure 3:**
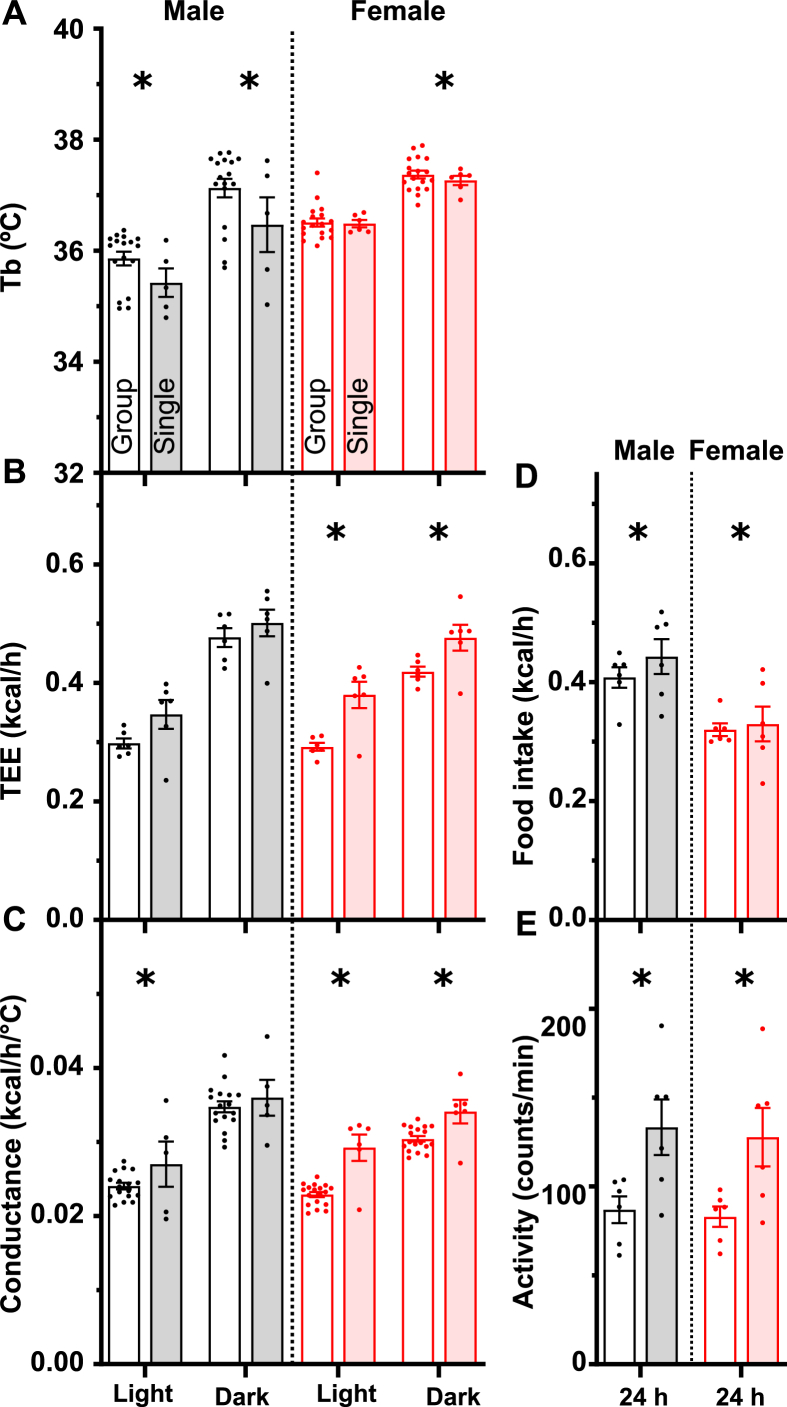
Effect of single housing on thermal physiology at 23 °C. Group-housed (no shading) and single-housed (with shading) mice were studied in a home-cage indirect calorimetry system. Each point is the mean of 12 h of measurements of (A) body temperature (Tb), (B) total energy expenditure (TEE), and (C) heat conductance, in males (black) and females (red) during the light or dark phase as indicated. (D) Food intake and (E) physical activity were averaged over 24 h. Data are mean ± SEM, n = 5–18/group. ∗ indicates a two-way ANOVA housing factor P < 0.05. Full statistical analysis, RER, and water intake are in Table S1.

Interestingly, at 23 °C, the Tb in single-housed males was slightly lower than in group-housed males (by 0.44 ± 0.14 °C (light) or 0.58 ± 0.21 °C (dark)), with no significant difference in TEE (Figure 3A and B, left panels; Table S1). In contrast, single-housed females retained a similar Tb to group-housed females by increasing TEE by 25.9 ± 4.6% (light) or 10.0 ± 3.3% (dark) (Figure 3A and B, right panels). The Tb results were replicated in an independent 3-day interval in the same mice (Table S1). Thus, male and female mice use different strategies to adjust their energy homeostasis between group and single housing.

Both TEE and Tb contribute to whole-body heat conductance, which was significantly higher in single-housed males during light (by 12.6 ± 5.2%), females during light (23.3 ± 2.7%), and females during dark (8.9 ± 2.5%) compared to group-housed mice (Figure 3C). Concordant with the TEE, food intake was higher with single housing (Figure 3D). Physical activity appeared higher in single housing, although coincident activity in group cages might be underestimated (Figure 3E). Taken together, these data demonstrate greater heat loss from single-housed mice, possibly due to an inability to huddle, with a lower Tb and/or greater TEE and food intake.

### 3.3 Thermal effects of single housing are amplified at low Ta

**Figure 4:**
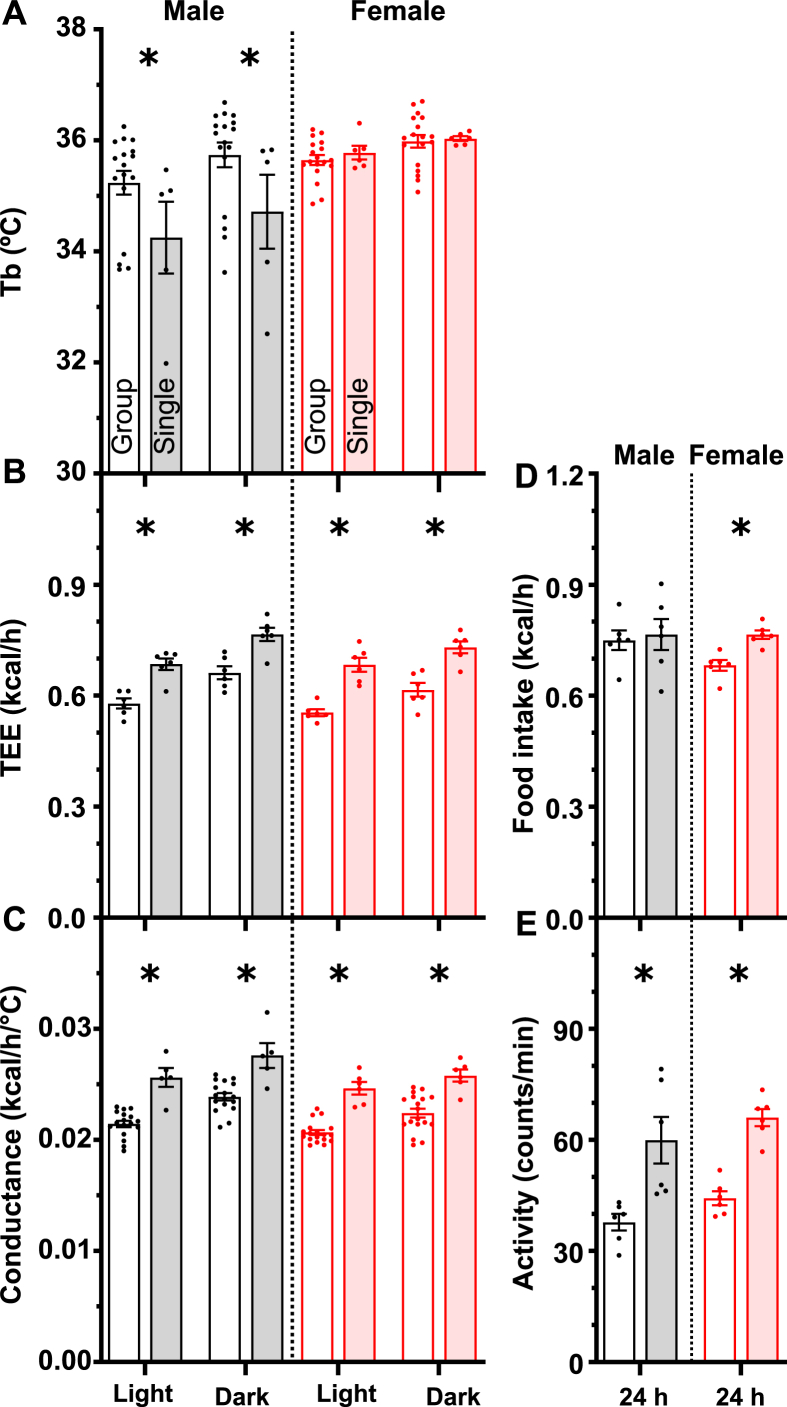
Effect of single housing on thermal physiology at 8 °C. Data from group- and single-housed mice are presented as described in the legend to Figure 3, except that 10 h of light phase data was used, avoiding the time during the Ta transition. ∗ indicates a two-way ANOVA housing factor P < 0.05. Additional data and statistical analysis are in Table S1.

We next examined the effect of a cold environment, 8 °C. Tb was lower in single-housed males by 0.74 ± 0.28 °C (light) and 0.83 ± 0.30 °C (dark), but unaffected in females (Figure 4A). In contrast, the TEE and whole-body heat conductance were higher with single housing in both males and females (Figure 4B and C). Food intake was increased at 8 °C (compared to 23 °C) and higher in single-housed females but not males, concordant with the TEE changes (Figure 4D; compare to Figure 3D). An 8 °C environment reduced physical activity in all groups, but activity remained greater in single-housed mice (Figure 4E). Thus single housing increases the thermal challenge posed by a cold environment. Single housing at 8 °C elicits the same sex-dimorphic changes that it does at room temperature, but the differences are quantitatively greater.

### 3.4 Single housing affects thermal physiology at a hot Ta

**Figure 5:**
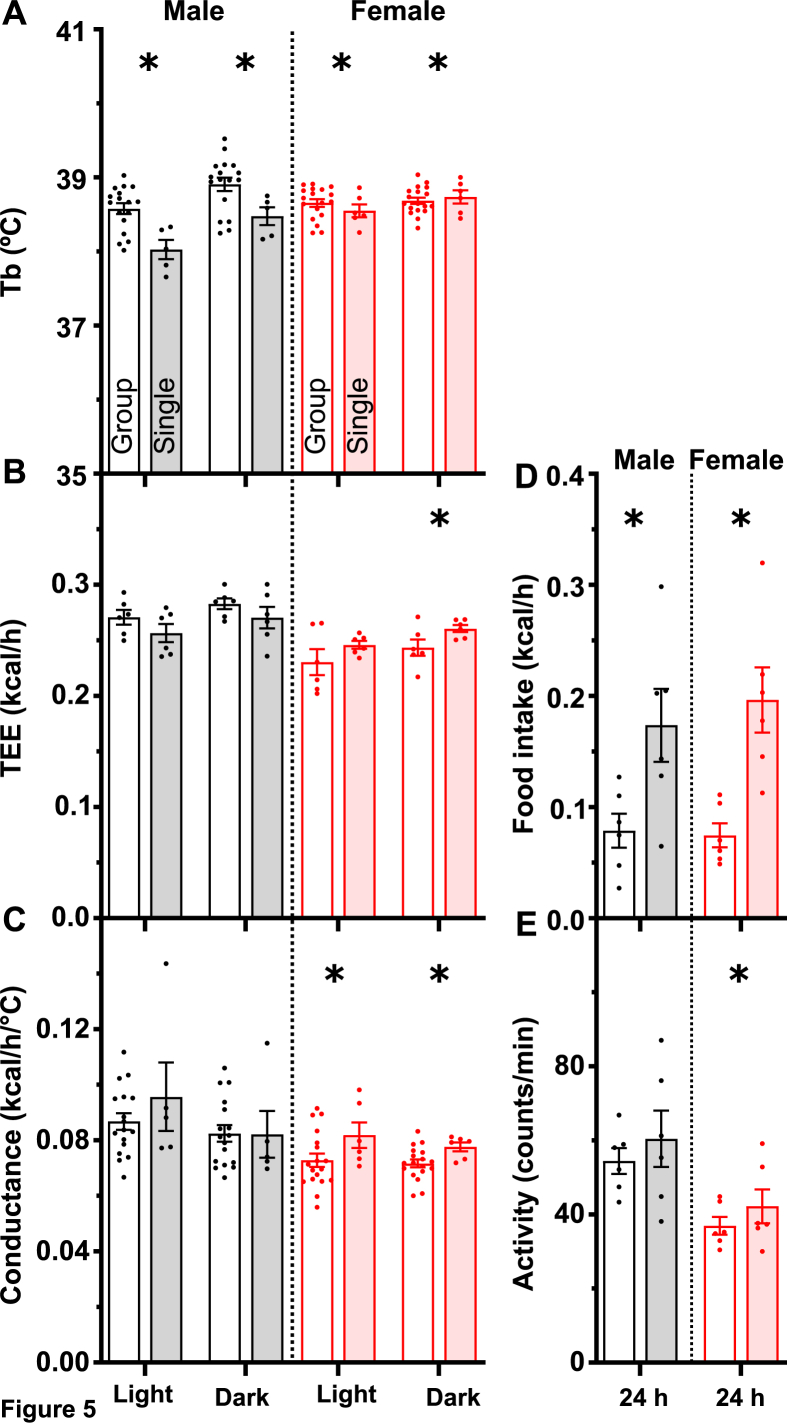
Effect of single housing on thermal physiology at 35 °C. Data from group- and single-housed mice are presented as described in the legend to Figure 3, except that 10 h of light phase data was used, avoiding the time during the Ta transition. ∗ indicates a two-way ANOVA housing factor P < 0.05. Additional data and statistical analysis are in Table S1.

The dark phase thermoneutral point (TNP_D_, ∼33 °C, the discrete Ta above which both TEE and Tb increase) defines a boundary between types of thermal physiology, with mice being heat stressed above this point [6]. Indeed, at a Ta of 35 °C, the Tb was ∼2.5 °C higher than that at a Ta of 23 °C, with a smaller increase in the single-housed mice (Figure 5A, compared to Figure 3A). TEE was reduced at 35 °C compared to cooler Tas, but was not majorly affected by housing density, likely because TEE was already minimized (Figure 5B). Heat conductance was increased in single-housed females, but not significantly in males (Figure 5C). At 35 °C, food intake was lower than at cooler Ta and lower in group-than in single-housed mice (Figure 5D). Physical activity was variable (Figure 5E). These results demonstrate that single housing allows better adaptation to a hot environment, exemplified by the Tb in males and heat conductance in females.

### 3.5 Fasting reduced Tb more in single-housed mice

**Figure 6:**
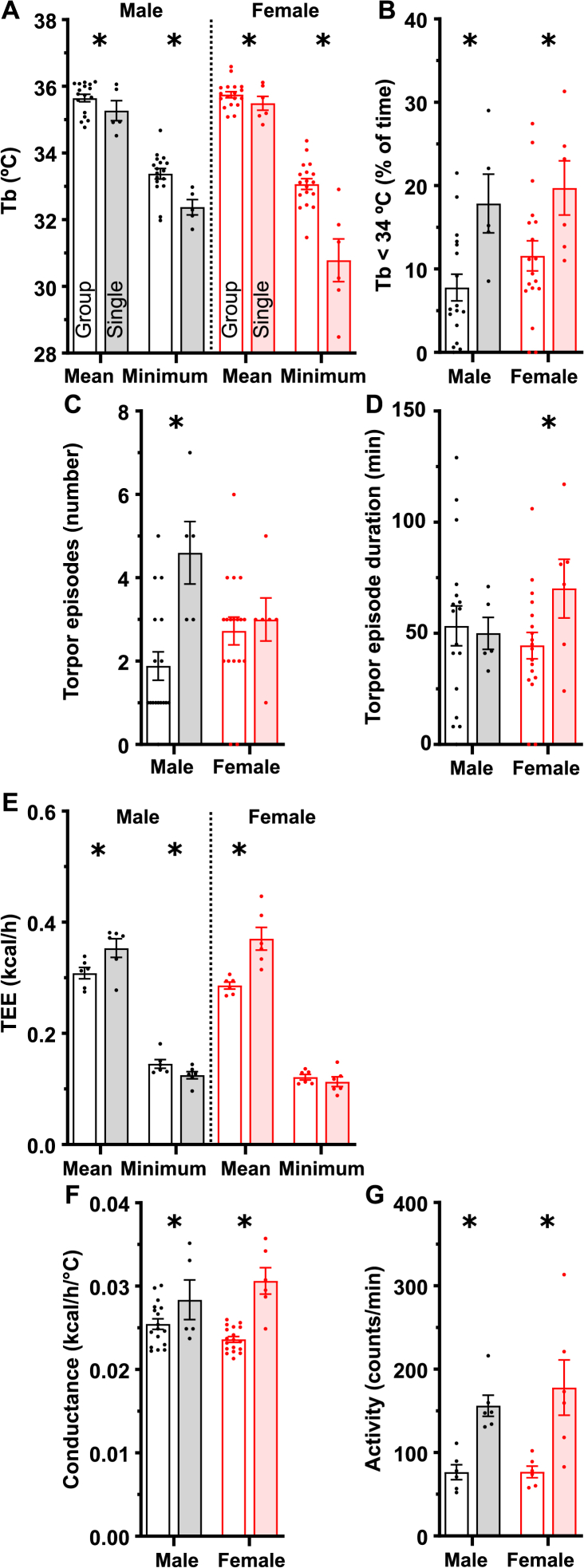
Effect of housing density on the response to a 24-h fast. Group- and single-housed mice were studied by indirect calorimetry at Ta ∼23 °C. (A) Mean and minimum Tb during the 24-h fast, (B) percentage of time with Tb < 34 °C, indicative of torpor, (C) number of torpor episodes (defined as the time when Tb drops below 34 °C to when it rises above 34 °C), (D) duration of torpor episodes, (E) total energy expenditure (TEE), (F) heat conductance, and (G) physical activity. Data are mean ± SEM, n = 5–18/group. ∗ indicates a two-way ANOVA housing factor P < 0.05. Full statistical analysis, RER, and water intake are in Table S1.

We next studied the effect of housing density on the response to a 24-h fast at 23 °C. The typical fasting response is an increase in physical activity followed by a mild reduction in Tb and/or torpor. Fasting of single-housed mice caused a greater drop in 24-h mean Tb and increased the percentage of time at which Tb was < 34 °C, classified as torpor (Figure 6A and B). Interestingly, this was due to an increase in the number of episodes of torpor in single-housed males, with no change in episode duration (Figure 6C and D). In contrast, the single-housed female mice showed no change in torpor episode numbers, but did show an increase in episode duration. 24-h mean TEE and heat conductance were increased in single-housed mice (Figure 6E and F). The minimum TEE was similar or lower in the single-housed mice, possibly because all three mice were not in torpor at the same time or because of a Q_10_ effect wherein the lower Tb reduces the TEE [33,34]. Physical activity was higher in single-housed mice (Figure 6G).

Similar Tb data were obtained in an independent study of the same mice using telemetry without indirect calorimetry (Table S1). The fasting results demonstrate that single- (vs group-) housed mice of both sexes have larger reductions in Tb, with male mice experiencing more torpor episodes of similar duration and female mice having a similar number of torpor episodes that last longer.

### 3.6 Heat conductance is decreasing, not constant, at cool Ta in group-housed mice

**Figure 7:**
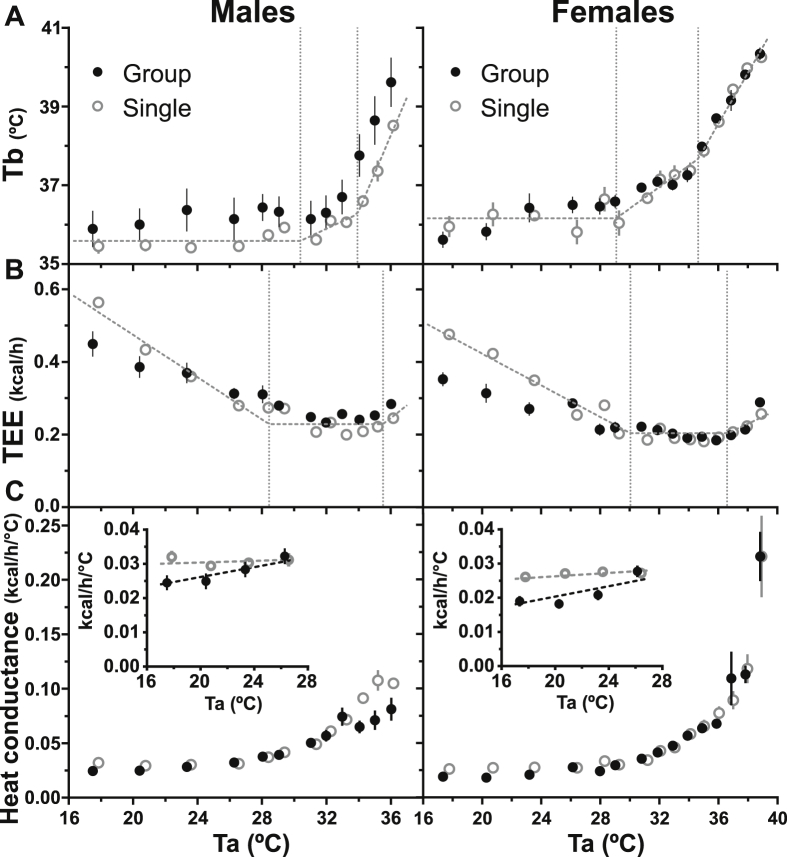
Scholander analysis of the effect of ambient temperature (Ta) on thermal physiology. (A) Body temperature (Tb), (B) total energy expenditure (TEE), and (C) heat conductance in group- and single-housed C57BL/6J mice. Data in A and B were analyzed by mixed-model segmented line regression, with the breakpoints indicated by the dotted vertical lines. The inserts in C expand the Ta < 27 °C region, including the linear regression lines. Statistical parameters are in Table S2. For visual clarity, only Ta plateau mean ± SE data points are depicted; however, all data (data shown plus data from transitions between Tas) were included in the regression models.

We next exposed independent cohorts of male and female C57BL/6J mice to short intervals of a wide range of Tas and analyzed as per Scholander [6,35]. To generalize the observations in 3.2, 3.3, and 3.4 (Figure 7A), male group-housed mice maintained a higher Tb, while females defended a similar Tb compared to their respective single-housed controls at all Ta. The single-housed mice showed the expected three-stage dependency of TEE and Tb on Ta, including linearly increasing TEE and constant heat conductance as Ta decreased below the light phase TNP (TNP_L_, the discrete Ta below which energy expenditure increases to defend Tb). However, group-housed mice of both sexes had decreasing heat conductance (lower compared to single-housed mice at 17 °C by 20% in males and 29% in females) and blunted TEE increases below thermoneutrality (Figure 7B and C, Table S2). Thus, the classic conclusions of the Scholander analysis, that TEE is linear and heat conductance is constant below the TNP_L_, while valid for single-housed mice, are not true for group-housed mice.

### 3.7 Single housing increased BAT *Ucp1* RNA expression

**Figure 8:**
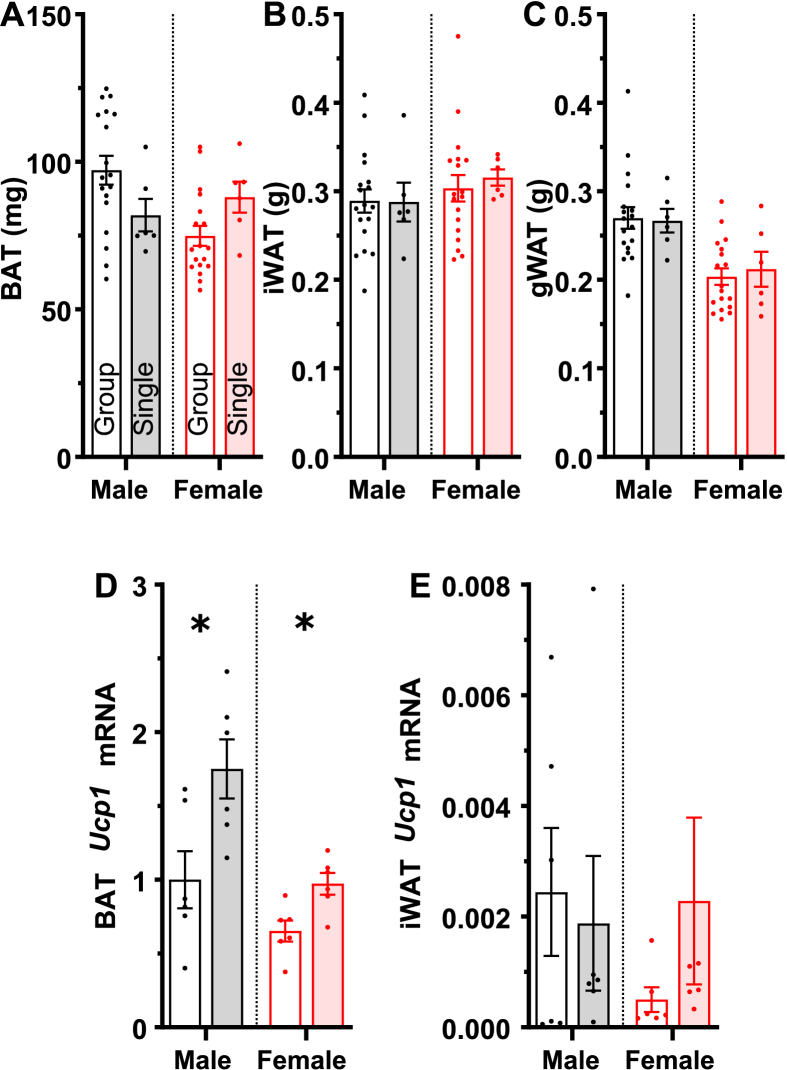
Effect of housing density on adipose tissue weight and *Ucp1* mRNA expression. Weight of (A) interscapular BAT, (B) iWAT, and (C) gWAT. *Ucp1* mRNA expression in (D) BAT and (E) iWAT, all normalized to male group-housed BAT. Mice were at 23 °C for at least four days when tissues were obtained. Data are mean ± SEM, n = 5–18/group. ∗ indicates a two-way ANOVA housing factor P < 0.05. Full statistical analysis is in Table S3.

To assess other effects of housing density, we studied mice at the end of four weeks of single vs group housing. In males, the single-housed mice weighed slightly less, with no difference in BAT, iWAT, or gWAT weight (Figure 8A–C). Leptin levels were lower, with no difference in thyroid hormones (T3 and T4). In female mice, housing density did not affect any of these parameters (Table S3).

In both sexes, BAT, but not iWAT, *Ucp1* mRNA levels were higher in the single-housed cohorts (Figure 8D and E). Increased *Ucp1* RNA demonstrates BAT adaptation to the increased thermogenic demand of single housing.

### 3.8 Effect of housing density on the *Brs3*-null phenotype

**Figure 9:**
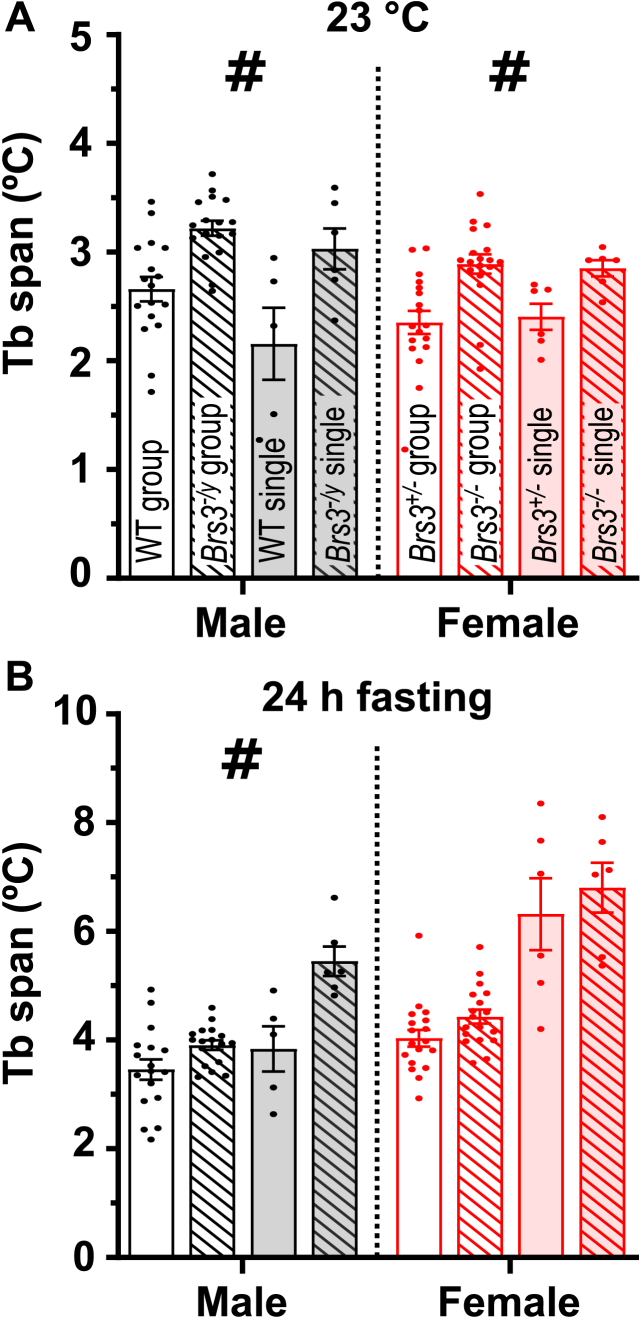
Effect of ambient temperature on the Tb span. The Tb span, the difference between the 95th and 5th percentiles of Tb over a 24-h interval, was measured (A) at 23 °C and (B) while fasting at 23 °C. Data are mean ± SEM, n = 5–18/group. # indicates genotype factor P < 0.05. Full statistical analysis is in Table S1.

Male *Brs3*-null mice have a mildly reduced resting light phase metabolic rate and Tb, a phenotype that is most robustly detected as an increased Tb span (see 2.4) [27,36]. The increased Tb span at 23 °C and with fasting were observed in both single and group housing (Figure 9A and B). The male *Brs3*-null mice also showed the expected small increase in body, BAT, iWAT, and gWAT weight, which were not affected by housing density (Table S3).

Female *Brs3*-null mice have not been studied previously. While slightly heavier at an earlier point (Figure S2), by the end of the study there was no difference in body, BAT, iWAT, or gWAT weight, nor were these affected by housing density. As in the males, the Tb span at 23 °C was increased in the female *Brs3*-null mice.

To screen for unexpected effects of housing density on the *Brs3*-null phenotype, we examined the ANOVA interaction term (housing × genotype). Overall, the frequency of P values < 0.05 was approximately that expected by chance (Table S1). However, the single-housed *Brs3*-null males had a lower fasting Tb than expected for additivity of the single housing and *Brs3*-null genotype effects. Thus, single housing amplified the *Brs3*-null phenotype compared to group housing.

## 4 Discussion

### 4.1 Housing density has major effects on thermal physiology

Mouse thermal physiology has been defined largely using single-housed mice. We extended these studies to group-housed mice. The single-housed mice are more affected by cold and fasting and less affected by a hot Ta, with different response mechanisms in male vs female mice (Figure S3). The lack of huddling, a form of social thermoregulation, by single-housed mice is a major contributor to the differences.

### 4.2 Single housing increases energy requirements in a cold environment

In the cold (i.e., below the TNP_L_), heat generation and preservation mechanisms are initiated, increasing energy expenditure to maintain Tb [6,35]. Group-housed mice can huddle, reducing their effective surface area and thus heat loss [37,38]. Single-housed mice cannot huddle, so to stay warm, they increase their energy expenditure (and food intake). Huddling reduced energy expenditure at 20 °C by ∼20%, with greater savings at lower Tas [39]. Huddling-driven reductions in heat conductance probably explain the Ta-dependent deviation of the group-housed TEE from the expected line in the Scholander analysis. Since mice huddle more during the light than the dark phase [40], the greater effect of single housing during the light phase is consistent with the quantitatively higher amount of huddling.

Insufficient food availability is a particular threat to mice due to their high mass-specific heat loss and metabolic rate; one adaptive response is torpor [41]. Torpor in single-housed mice reached lower Tbs than in group-housed mice. Thus the reduced ability to conserve heat (greater heat conductance) in single housing amplifies the effects of both fasting and cold exposure.

### 4.3 Single housing is beneficial in hot environments

Mice have a limited ability to dissipate energy by evaporative and dry heat loss [42,43], and at hot temperatures (i.e., above the TNP_D_), their heat loss mechanisms are overwhelmed [6]. We found that the group-housed mice were under more severe heat stress, evidenced by their higher Tb. Two mechanisms likely contribute to the increased heat stress in group-housed mice. First, the relative humidity was higher for the group-housed mice, limiting evaporative heat loss. Second, any huddling by group-housed mice would impair heat loss. Thus single-housing can aid coping with a hot environment in multiple ways.

### 4.4 Sex differences in the effects of single housing on thermal physiology

Tb is regulated in a sex-dimorphic pattern, with much of the differential regulation driven by estrogens (reviewed in [44]). Brain estrogen receptor expression varies by sex, including in regions that control Tb [21,45]. In females, Tb is generally higher, fluctuating with the estrous cycle [20]. Another sex difference is that female mice huddle more than male mice at room temperature, while the two sexes huddle similarly in the cold [40]. We found a sex-dimorphic response to single housing in cool environments, with female mice increasing TEE to defend the same Tb as group-housed mice. In contrast, the defended Tb in single-housed males was lower than in group-housed mice.

The thermoregulatory response to fasting also depends on sex. Single housing caused more fasting-induced torpor in both sexes, but through different mechanisms. The males increased the number of torpor episodes, while the females increased episode duration instead. These results are consistent with studies showing sex differences in torpor features in mice [21] and other species [46]. Thus, there are multiple sex-specific differences in the effects of housing density on thermal physiology.

### 4.5 Limited effect of housing density on the thermal phenotype of *Brs3*-null mice

*Brs3*-null mice were used as a test case to assess whether single housing potentiates or masks a subtle thermal phenotype. Prior studies of *Brs3*-null mice have exclusively studied males. Here, we also studied female mice and found that they have a larger Tb span, but less body weight increase on a chow diet. One limitation is that the female controls were heterozygotes, not homozygous wild type.

We draw two conclusions from the *Brs3*-null mice. First, there is little evidence for non-additive housing × genotype interactions (qualitative effects)—group housing did not expose novel thermal physiology caused by the loss of *Brs3* that was not detected by single housing. Second, single housing appears to be quantitatively more sensitive than group housing in screening mutant mice for thermal phenotypes. In retrospect, the *Brs3*-null mice are also a validation cohort for the results obtained in the wild type mice.

### 4.6 Use of single housing to study thermal physiology

National Research Council laboratory animal guidelines suggest that single housing should be avoided whenever possible [47], with the implicit assumption that it is stressful. However, the experimental support for this is conflicting, with lower [[48], [49], [50]], similar [51], or higher [52,53] levels of stress in single-housed male mice, as measured by corticosterone levels and behavioral assays. Even in females, which are less territorial [54], increased stress in single-housed mice was found by some authors [50] but not others [51]. It seems that group housing has not been unequivocally demonstrated to be less stressful, but is more stressful from a thermoregulatory standpoint in hot environments. In addition, our results demonstrate a clear advantage for using single housing to study thermal physiology by increasing the dynamic ranges. The lack of huddling removes a major confounding behavioral effect. The larger effect sizes mean that experiments can be smaller, with fewer mice needed to adequately power experiments.

In additional to diurnal patterning, physical activity on shorter time scales is distributed between inactive and active periods, with the latter having higher Tb and energy expenditure. The timing of these periods is random [55], but is partially synchronized when mice are group housed. Thus single housing is useful when studying patterns of activity.

Importantly, the effects of housing are not limited to quantitative effects. Group-housed mice decrease their heat conductance with decreasing ambient temperature below 27 °C, while single-housed mice do not. The group-housed mice violate the energy expenditure vs ambient temperature relationship described by Scholander [35], precluding insights such as determination of the defended body temperature. Thus single housing reveals principles of thermal physiology that are masked by group housing.

### 4.7 Housing density and body weight

We did not see an effect of housing density on body weight. There are conflicting results regarding the effects of single housing on body weight [56], with an increased body weight with single housing being more likely if the mice were separated at a young age [11]. Our mice were single housed for 5 weeks, starting at 10 or 15 weeks of age. If housing density affects body weight, the magnitude is modest and/or confounded by other factors such as age. Longer experiments with larger cohort sizes are needed to investigate this further.

### 4.8 Conclusions

Thermal physiology is different in single-versus group-housed mice, with different strategies used in male and female mice. Thermal physiology is fundamentally different between mice and humans; the current findings inform interpretation of mouse studies and their applicability to human physiology. Single housing aids investigation of thermal physiology because it precludes thermal insulation caused by huddling, reduces the thermal stress of a hot environment, and amplifies the effects of fasting or a cold environment.

## Data availability statement

The data generated during this study are available for download at Open Science Framework: https://osf.io/34pes/.
