# Supplementary material for: The effects of housing density on mouse thermal physiology depend on sex and ambient temperature
Source: Mol Metab. 2021 Sep 1;53:101332. doi: 10.1016/j.molmet.2021.101332 (PMC8463779; doi:10.1016/j.molmet.2021.101332)
Supplement: Multimedia component 1 [file mmc1.pdf]

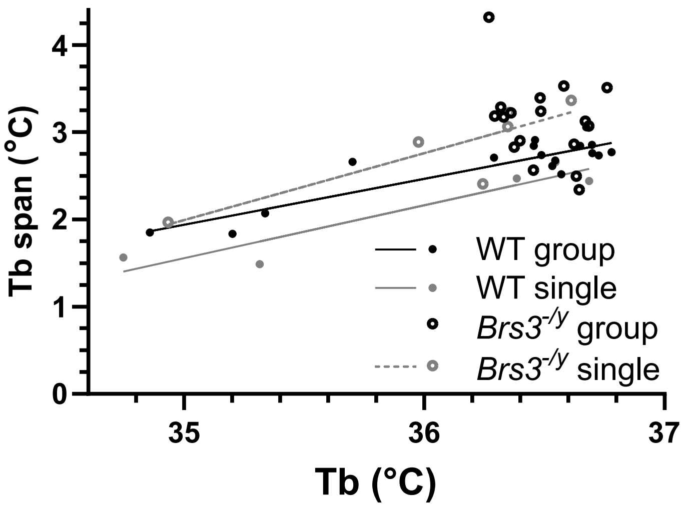

Figure S1: Tb span dependence on Tb. Using 24-hour measurement periods, the Tb span (the 95th percentile minus the 5th percentile) and 24-h mean Tb were analyzed by linear regression in the indicated groups of male mice at 23 °C. The regression lines are: WT group, Tb span=0.5271*Tb-16.51, R^2 = 0.80; WT single, Tb span=0.6048*Tb-19.61, R^2 = 0.88; *Brs3$^{-/y}$* group, R^2 < 0.1, and *Brs$^{3-/y}$* single, Tb span=0.7672*Tb-24.86, R^2 = 0.74. The regression line slopes are different from zero at P<0.0001, P=0.018, P=0.22, and P=0.028, respectively.

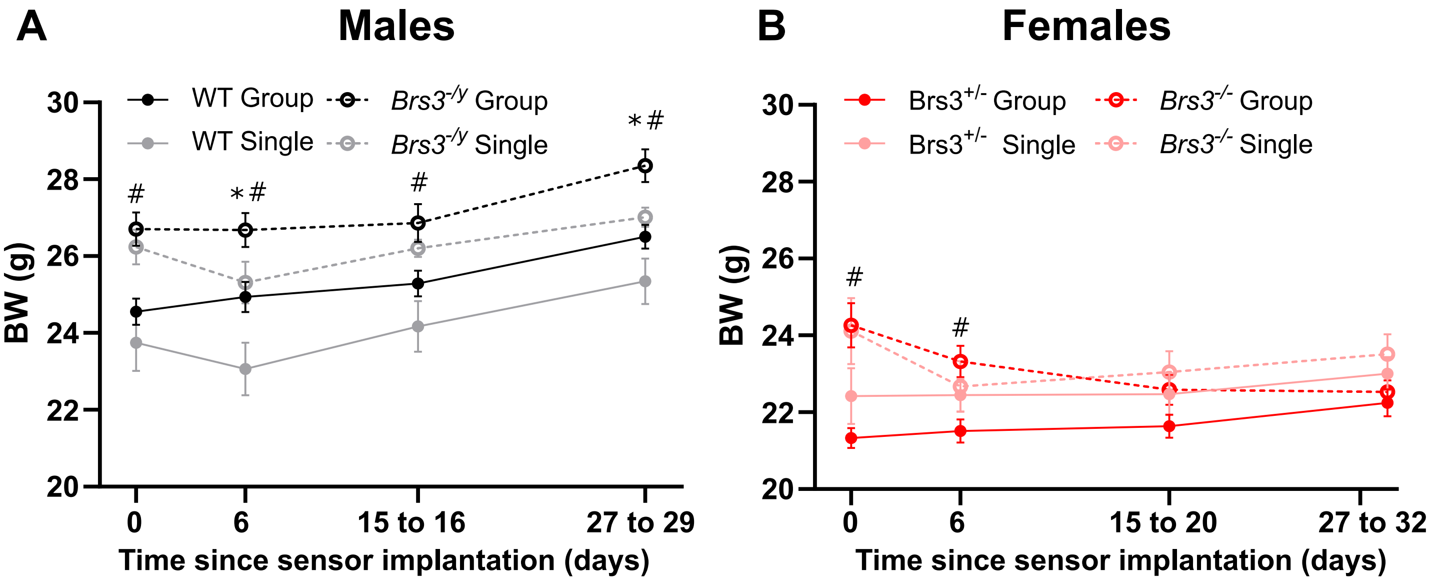

Figure S2: Body weights during study. Body weights were measured on the indicated day after telemeter implantation in (A) male mice implanted at 10 weeks and (B) female mice implanted at 15 weeks of age. 2-way ANOVA: * $P < 0.05$ effect of housing, # $P < 0.05$ effect of genotype, n = 5 – 18/group.

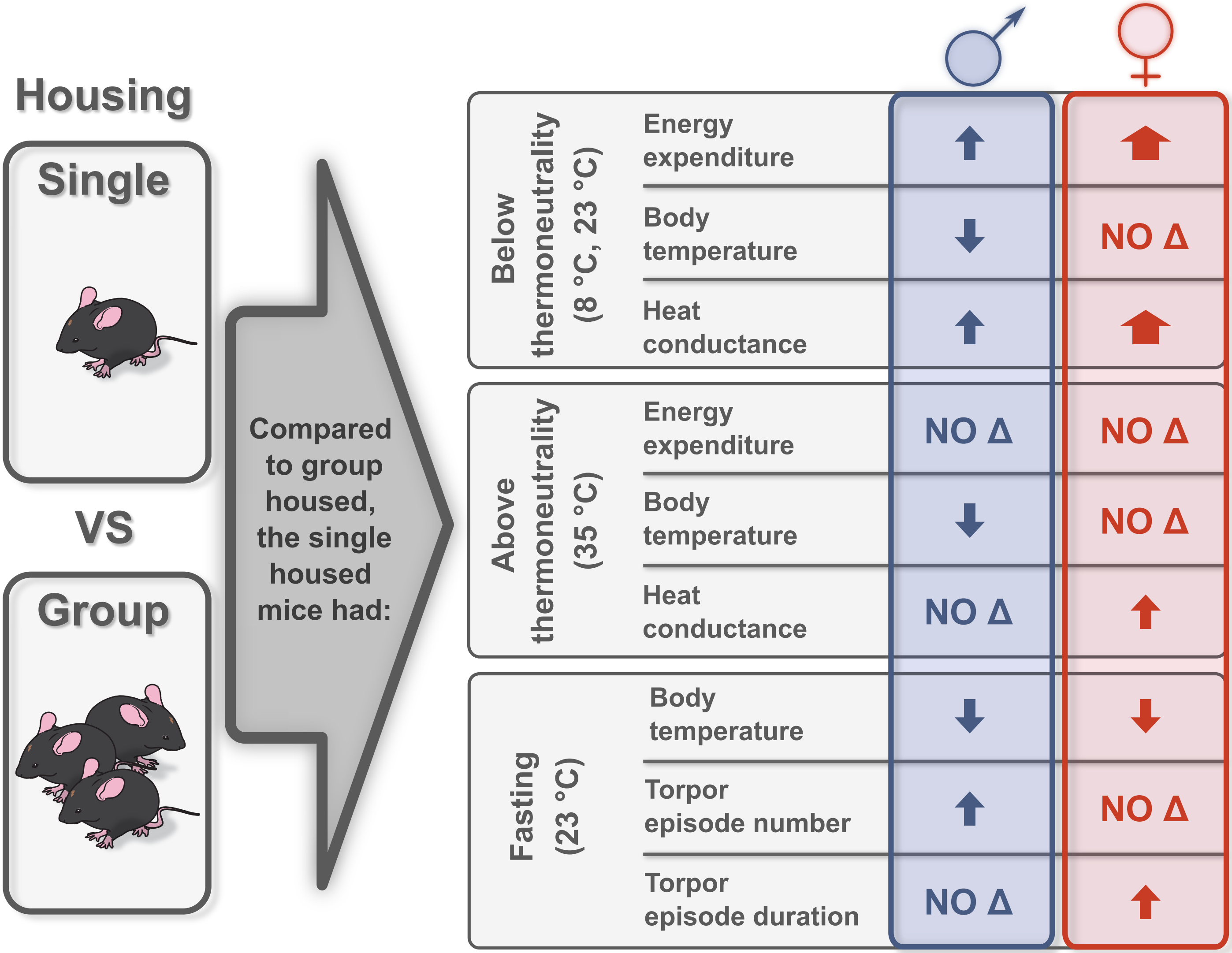

Figure S3: Thermal physiology is different in single versus group housed mice, with different strategies used in male and female mice.

Table S1: Summary of measured parameters

	Males									Females								
	WT group	WT single	WT *P* value*	Brs3-/y group	Brs3-/y single	Brs3-/y *P* value	2-way ANOVA *P* value housing	genotype	interaction	Brs3+/- group	Brs3+/- single	Brs3+/- *P* value	Brs3-/- group	Brs3-/- single	Brs3-/- *P* value	2-way ANOVA *P* value housing	genotype	interaction
**Tb telemetry (section 3.2)**																		
Light Tb (°C)	35.76 ± 0.12	35.51 ± 0.30	0.47	35.88 ± 0.05	35.57 ± 0.21	0.27	0.08	0.56	0.84	36.27 ± 0.06	36.34 ± 0.13	0.85	36.08 ± 0.07	36.04 ± 0.10	0.95	0.88	0.0134	0.57
Dark Tb (°C)	36.84 ± 0.18	36.41 ± 0.47	0.34	37.13 ± 0.04	36.65 ± 0.28	0.22	0.0438	0.23	0.91	37.11 ± 0.09	37.28 ± 0.15	0.47	37.32 ± 0.05	36.89 ± 0.14	0.0114	0.21	0.40	0.0068
Dark Tb - light Tb (°C)	1.08 ± 0.07	0.90 ± 0.17	0.44	1.25 ± 0.07	1.08 ± 0.14	0.44	0.11	0.10	0.95	0.84 ± 0.08	0.94 ± 0.07	0.72	1.24 ± 0.05	0.85 ± 0.13	0.0101	0.12	0.11	0.0126
24 h Tb (°C)	36.27 ± 0.15	35.94 ± 0.38	0.37	36.49 ± 0.04	36.08 ± 0.24	0.19	0.0424	0.32	0.83	36.64 ± 0.07	36.76 ± 0.14	0.62	36.63 ± 0.06	36.44 ± 0.11	0.30	0.71	0.09	0.11
Tb span (°C)	2.61 ± 0.09	2.12 ± 0.25	0.07	3.12 ± 0.11	2.82 ± 0.22	0.29	0.0140	0.0004	0.54	2.95 ± 0.08	2.58 ± 0.12	0.0370	3.24 ± 0.08	3.03 ± 0.11	0.29	0.0089	0.0013	0.48
**23 °C Ambient temperature (Figure 3)**																		
Light TEE (kcal/h)	0.298 ± 0.008	0.347 ± 0.024	0.26	0.333 ± 0.022	0.358 ± 0.030	0.69	0.11	0.31	0.60	0.292 ± 0.007	0.380 ± 0.022	0.0004	0.300 ± 0.005	0.365 ± 0.014	0.0055	<0.0001	0.79	0.43
Dark TEE (kcal/h)	0.477 ± 0.016	0.502 ± 0.023	0.76	0.494 ± 0.024	0.501 ± 0.037	0.97	0.54	0.75	0.75	0.419 ± 0.008	0.476 ± 0.022	0.0236	0.456 ± 0.010	0.486 ± 0.014	0.29	0.0071	0.13	0.37
24 h TEE (kcal/h)	0.388 ± 0.011	0.424 ± 0.023	0.49	0.414 ± 0.023	0.430 ± 0.033	0.87	0.28	0.52	0.67	0.356 ± 0.006	0.428 ± 0.021	0.0016	0.378 ± 0.004	0.426 ± 0.013	0.0318	0.0002	0.46	0.36
Light Tb (°C)	35.86 ± 0.12	35.42 ± 0.26	0.08	35.87 ± 0.04	35.42 ± 0.17	0.05	0.0033	1.00	0.97	36.51 ± 0.07	36.49 ± 0.06	0.98	36.26 ± 0.07	36.08 ± 0.09	0.34	0.29	0.0011	0.41
Dark Tb (°C)	37.13 ± 0.16	36.47 ± 0.49	0.07	37.25 ± 0.05	36.75 ± 0.24	0.16	0.0079	0.34	0.70	37.37 ± 0.07	37.27 ± 0.08	0.65	37.55 ± 0.07	37.11 ± 0.07	0.0025	0.0041	0.91	0.07
Dark Tb - light Tb (°C)	1.27 ± 0.07	1.04 ± 0.27	0.34	1.38 ± 0.07	1.33 ± 0.11	0.92	0.23	0.09	0.46	0.86 ± 0.08	0.78 ± 0.13	0.83	1.29 ± 0.07	1.03 ± 0.10	0.17	0.12	0.0028	0.41
24 h Tb (°C)	36.48 ± 0.13	35.96 ± 0.38	0.07	36.58 ± 0.04	36.08 ± 0.20	0.07	0.0037	0.52	0.94	36.92 ± 0.06	36.86 ± 0.04	0.82	36.85 ± 0.06	36.53 ± 0.08	0.0108	0.0178	0.0169	0.10
Tb span (°C)	2.66 ± 0.11	2.16 ± 0.33	0.06	3.22 ± 0.07	3.03 ± 0.19	0.61	0.0311	<0.0001	0.32	2.35 ± 0.11	2.41 ± 0.12	0.94	2.89 ± 0.09	2.85 ± 0.08	0.97	0.95	0.0004	0.71
Light conductance (kcal/h/Δ°C)	0.024 ± 0.000	0.027 ± 0.003	0.25	0.026 ± 0.001	0.029 ± 0.002	0.13	0.0204	0.13	0.87	0.023 ± 0.000	0.029 ± 0.002	<0.0001	0.024 ± 0.000	0.028 ± 0.001	<0.0001	<0.0001	0.93	0.17
Dark conductance (kcal/h/Δ°C)	0.035 ± 0.001	0.036 ± 0.002	0.79	0.035 ± 0.001	0.037 ± 0.002	0.53	0.2582	0.83	0.80	0.030 ± 0.000	0.034 ± 0.002	0.0039	0.033 ± 0.001	0.035 ± 0.001	0.17	0.0009	0.05	0.28
Light RER	0.843 ± 0.011	0.784 ± 0.025	0.07	0.838 ± 0.008	0.784 ± 0.023	0.10	0.0059	0.92	0.88	0.836 ± 0.014	0.820 ± 0.011	0.71	0.831 ± 0.017	0.796 ± 0.015	0.21	0.10	0.33	0.52
Dark RER	0.908 ± 0.006	0.879 ± 0.023	0.37	0.918 ± 0.002	0.878 ± 0.020	0.16	0.0388	0.77	0.73	0.900 ± 0.006	0.874 ± 0.019	0.35	0.888 ± 0.014	0.874 ± 0.013	0.75	0.17	0.65	0.65
Light food intake (kcal/h)	0.171 ± 0.031	0.125 ± 0.036	0.55	0.181 ± 0.025	0.210 ± 0.037	0.78	0.80	0.16	0.26	0.136 ± 0.030	0.219 ± 0.040	0.11	0.138 ± 0.019	0.190 ± 0.021	0.38	0.0292	0.66	0.60
Dark food intake (kcal/h)	0.645 ± 0.015	0.762 ± 0.052	0.06	0.672 ± 0.012	0.758 ± 0.047	0.20	0.0108	0.76	0.67	0.505 ± 0.025	0.489 ± 0.042	0.93	0.461 ± 0.038	0.587 ± 0.034	0.0387	0.13	0.44	0.06
24 h food intake (kcal/h)	0.408 ± 0.017	0.443 ± 0.029	0.45	0.426 ± 0.010	0.484 ± 0.024	0.13	0.0408	0.19	0.60	0.320 ± 0.011	0.330 ± 0.029	0.94	0.300 ± 0.022	0.389 ± 0.022	0.0191	0.0367	0.40	0.09
Light water intake (ml/h)	0.078 ± 0.007	0.079 ± 0.016	>0.99	0.077 ± 0.013	0.099 ± 0.012	0.40	0.38	0.46	0.39	0.066 ± 0.018	0.125 ± 0.016	0.0402	0.070 ± 0.008	0.125 ± 0.022	0.06	0.0027	0.90	0.89
Dark water intake (ml/h)	0.250 ± 0.019	0.315 ± 0.036	0.72	0.244 ± 0.019	0.310 ± 0.020	0.13	0.07	0.87	0.41	0.206 ± 0.023	0.211 ± 0.034	0.99	0.204 ± 0.018	0.325 ± 0.018	0.0045	0.0179	0.0323	0.0282
24 h water intake (ml/h)	0.164 ± 0.009	0.197 ± 0.023	0.26	0.160 ± 0.012	0.204 ± 0.012	0.10	0.0187	0.91	0.71	0.136 ± 0.008	0.168 ± 0.018	0.22	0.137 ± 0.007	0.225 ± 0.018	0.0005	0.0003	0.05	0.06
Light PA (counts/min)	42 ± 5	66 ± 14	0.81	45 ± 5	125 ± 55	0.12	0.08	0.29	0.33	45 ± 3	73 ± 7	0.0051	59 ± 6	63 ± 6	0.80	0.0096	0.74	0.06
Dark PA (counts/min)	131 ± 11	201 ± 19	0.0025	110 ± 5	163 ± 14	0.0199	0.0001	0.0356	0.53	121 ± 9	182 ± 27	0.0175	145 ± 7	189 ± 6	0.10	0.0022	0.32	0.56
24 h PA (counts/min)	87 ± 7	133 ± 16	0.0197	77 ± 5	118 ± 14	0.0441	0.0012	0.29	0.79	83 ± 6	128 ± 16	0.0051	102 ± 4	126 ± 4	0.14	0.0012	0.36	0.28
**8 °C Ambient temperature (Figure 4)**																		
Light TEE (kcal/h)	0.579 ± 0.014	0.685 ± 0.016	0.0197	0.613 ± 0.015	0.703 ± 0.046	0.05	0.0013	0.33	0.76	0.554 ± 0.009	0.683 ± 0.019	<0.0001	0.566 ± 0.003	0.689 ± 0.028	0.0002	<0.0001	0.63	0.87
Dark TEE (kcal/h)	0.662 ± 0.018	0.766 ± 0.018	0.0266	0.720 ± 0.018	0.772 ± 0.044	0.34	0.0093	0.25	0.35	0.616 ± 0.019	0.730 ± 0.016	0.0009	0.633 ± 0.010	0.722 ± 0.028	0.0079	<0.0001	0.82	0.51
24 h TEE (kcal/h)	0.624 ± 0.015	0.729 ± 0.017	0.0196	0.671 ± 0.016	0.741 ± 0.044	0.14	0.0032	0.27	0.50	0.588 ± 0.014	0.709 ± 0.017	0.0002	0.603 ± 0.006	0.707 ± 0.028	0.0011	<0.0001	0.73	0.64
Light Tb (°C)	35.24 ± 0.21	34.25 ± 0.64	0.0371	35.30 ± 0.08	34.80 ± 0.32	0.35	0.0102	0.28	0.38	35.65 ± 0.09	35.78 ± 0.12	0.74	35.71 ± 0.12	35.63 ± 0.11	0.89	0.85	0.75	0.44
Dark Tb (°C)	35.74 ± 0.22	34.71 ± 0.67	0.0446	35.94 ± 0.09	35.32 ± 0.40	0.24	0.0078	0.18	0.51	35.99 ± 0.11	36.03 ± 0.04	0.96	35.91 ± 0.10	35.78 ± 0.12	0.76	0.77	0.24	0.53
Dark Tb - light Tb (°C)	0.50 ± 0.07	0.46 ± 0.10	0.96	0.65 ± 0.05	0.52 ± 0.14	0.53	0.38	0.30	0.62	0.34 ± 0.08	0.25 ± 0.13	0.84	0.20 ± 0.10	0.15 ± 0.07	0.95	0.57	0.31	0.86
24 h Tb (°C)	35.52 ± 0.22	34.52 ± 0.66	0.0412	35.64 ± 0.08	35.09 ± 0.36	0.29	0.0092	0.23	0.44	35.83 ± 0.09	35.90 ± 0.08	0.93	35.81 ± 0.10	35.69 ± 0.11	0.74	0.83	0.41	0.47
Tb span (°C)	2.47 ± 0.09	2.21 ± 0.22	0.34	2.96 ± 0.09	2.40 ± 0.11	0.0086	0.0042	0.015	0.28	2.56 ± 0.19	2.47 ± 0.22	0.96	2.90 ± 0.21	2.39 ± 0.21	0.32	0.25	0.63	0.43
Light conductance (kcal/h/Δ°C)	0.021 ± 0.000	0.026 ± 0.001	<0.0001	0.022 ± 0.000	0.026 ± 0.001	0.0003	<0.0001	0.39	0.61	0.021 ± 0.000	0.025 ± 0.001	<0.0001	0.021 ± 0.000	0.025 ± 0.001	<0.0001	<0.0001	0.86	0.90
Dark conductance (kcal/h/Δ°C)	0.024 ± 0.000	0.028 ± 0.001	0.0011	0.025 ± 0.000	0.028 ± 0.001	0.0471	<0.0001	0.24	0.26	0.022 ± 0.000	0.026 ± 0.001	<0.0001	0.023 ± 0.000	0.026 ± 0.001	0.0012	<0.0001	0.89	0.57
Light RER	0.879 ± 0.006	0.861 ± 0.011	0.26	0.869 ± 0.005	0.850 ± 0.009	0.25	0.041	0.21	0.99	0.906 ± 0.006	0.907 ± 0.014	1.00	0.911 ± 0.005	0.897 ± 0.006	0.45	0.44	0.77	0.39
Dark RER	0.915 ± 0.008	0.889 ± 0.013	0.16	0.912 ± 0.005	0.889 ± 0.012	0.22	0.024	0.87	0.91	0.946 ± 0.004	0.951 ± 0.011	0.88	0.937 ± 0.005	0.926 ± 0.011	0.59	0.73	0.06	0.34
Light food intake (kcal/h)	0.683 ± 0.048	0.742 ± 0.035	0.56	0.640 ± 0.055	0.646 ± 0.024	0.99	0.45	0.11	0.54	0.632 ± 0.028	0.794 ± 0.040	0.0049	0.593 ± 0.043	0.752 ± 0.014	0.0058	<0.0001	0.23	0.96
Dark food intake (kcal/h)	0.806 ± 0.034	0.786 ± 0.057	0.96	0.817 ± 0.071	0.947 ± 0.049	0.21	0.33	0.13	0.19	0.676 ± 0.054	0.741 ± 0.032	0.49	0.741 ± 0.021	0.731 ± 0.053	0.98	0.52	0.52	0.38
24 h food intake (kcal/h)	0.750 ± 0.027	0.766 ± 0.042	0.94	0.736 ± 0.032	0.810 ± 0.036	0.28	0.21	0.66	0.42	0.656 ± 0.031	0.765 ± 0.011	0.05	0.674 ± 0.030	0.740 ± 0.034	0.13	0.0062	0.51	0.74
Light water intake (ml/h)	0.111 ± 0.028	0.198 ± 0.054	0.18	0.144 ± 0.019	0.162 ± 0.029	0.92	0.15	0.98	0.34	0.156 ± 0.022	0.161 ± 0.046	0.99	0.159 ± 0.015	0.283 ± 0.013	0.0094	0.029	0.0341	0.0446
Dark water intake (ml/h)	0.214 ± 0.025	0.242 ± 0.041	0.72	0.186 ± 0.023	0.261 ± 0.009	0.13	0.07	0.87	0.41	0.167 ± 0.011	0.135 ± 0.035	0.66	0.161 ± 0.023	0.244 ± 0.030	0.07	0.34	0.07	0.0431
24 h water intake (ml/h)	0.157 ± 0.020	0.203 ± 0.036	0.33	0.167 ± 0.018	0.216 ± 0.014	0.30	0.06	0.64	0.96	0.162 ± 0.013	0.147 ± 0.039	0.90	0.160 ± 0.017	0.261 ± 0.021	0.0171	0.09	0.0328	0.0289
Light PA (counts/min)	32 ± 3	44 ± 5	0.08	31 ± 2	45 ± 5	0.05	0.0039	0.94	0.88	40 ± 1	63 ± 4	0.0187	36 ± 2	57 ± 10	0.0296	0.0009	0.36	0.88
Dark PA (counts/min)	43 ± 2	73 ± 10	0.0140	49 ± 1	77 ± 10	0.0291	0.0007	0.48	0.82	48 ± 4	69 ± 3	0.0036	49 ± 2	65 ± 6	0.0199	0.0002	0.78	0.60
24 h PA (counts/min)	38 ± 2	60 ± 6	0.0070	41 ± 1	62 ± 7	0.0107	0.0002	0.58	0.89	44 ± 2	66 ± 2	0.0030	43 ± 2	61 ± 8	0.0102	0.0001	0.48	0.71
**35 °C Ambient temperature (Figure 5)**																		
Light TEE (kcal/h)	0.271 ± 0.007	0.257 ± 0.008	0.54	0.288 ± 0.015	0.265 ± 0.008	0.20	0.07	0.21	0.64	0.231 ± 0.012	0.246 ± 0.004	0.34	0.244 ± 0.010	0.240 ± 0.004	0.93	0.48	0.64	0.25
Dark TEE (kcal/h)	0.283 ± 0.005	0.270 ± 0.010	0.73	0.307 ± 0.015	0.306 ± 0.016	1.00	0.60	0.0253	0.64	0.244 ± 0.007	0.261 ± 0.003	0.20	0.256 ± 0.007	0.277 ± 0.010	0.09	0.0136	0.05	0.77
24 hTEE (kcal/h)	0.277 ± 0.005	0.264 ± 0.009	0.61	0.298 ± 0.014	0.287 ± 0.012	0.70	0.25	0.0470	0.92	0.238 ± 0.009	0.254 ± 0.002	0.21	0.251 ± 0.008	0.261 ± 0.006	0.53	0.07	0.18	0.66
Light Tb (°C)	38.58 ± 0.07	38.03 ± 0.13	0.0089	38.39 ± 0.10	37.86 ± 0.18	0.0071	0.0001	0.16	0.93	38.66 ± 0.05	38.55 ± 0.09	0.67	38.57 ± 0.08	37.96 ± 0.12	<0.0001	0.0003	0.0006	0.0085
Dark Tb (°C)	38.91 ± 0.09	38.48 ± 0.12	0.0326	38.86 ± 0.06	38.69 ± 0.19	0.53	0.0154	0.48	0.27	38.69 ± 0.04	38.74 ± 0.09	0.87	38.70 ± 0.06	38.31 ± 0.09	0.0016	0.0320	0.0110	0.0061
Dark Tb - light Tb (°C)	0.33 ± 0.03	0.45 ± 0.08	0.43	0.47 ± 0.05	0.83 ± 0.14	0.0010	0.0013	0.0006	0.09	0.03 ± 0.05	0.18 ± 0.10	0.30	0.14 ± 0.05	0.35 ± 0.11	0.10	0.0199	0.08	0.68
24 h Tb (°C)	38.75 ± 0.08	38.28 ± 0.12	0.0196	38.64 ± 0.08	38.28 ± 0.19	0.06	0.0012	0.63	0.64	38.66 ± 0.04	38.66 ± 0.07	1.00	38.64 ± 0.07	38.14 ± 0.08	<0.0001	0.0017	0.0011	0.0019
Tb span (°C)	1.20 ± 0.08	1.40 ± 0.17	0.59	3	2.07 ± 0.30	0.0026	0.0041	0.0079	0.10	1.15 ± 0.08	1.07 ± 0.08	0.80	1.10 ± 0.06	1.40 ± 0.07	0.0396	0.20	0.12	0.0392
Light conductance (kcal/h/Δ°C)	0.087 ± 0.003	0.096 ± 0.012	0.49	0.092 ± 0.004	0.105 ± 0.006	0.21	0.07	0.20	0.75	0.073 ± 0.002	0.082 ± 0.005	0.10	0.083 ± 0.002	0.091 ± 0.004	0.14	0.0096	0.0059	0.92
Dark conductance (kcal/h/Δ°C)	0.083 ± 0.003	0.082 ± 0.008	1.00	0.086 ± 0.004	0.092 ± 0.004	0.61	0.57	0.15	0.52	0.072 ± 0.001	0.078 ± 0.002	0.11	0.079 ± 0.002	0.089 ± 0.002	0.0026	0.0004	<0.0001	0.31
Light RER	0.811 ± 0.012	0.763 ± 0.017	0.06	0.842 ± 0.017	0.775 ± 0.011	0.0079	0.0008	0.15	0.52	0.829 ± 0.007	0.835 ± 0.036	0.97	0.882 ± 0.020	0.843 ± 0.004	0.41	0.47	0.19	0.32
Dark RER	0.804 ± 0.021	0.746 ± 0.019	0.10	0.830 ± 0.017	0.776 ± 0.023	0.14	0.0111	0.18	0.90	0.828 ± 0.021	0.853 ± 0.025	0.60	0.833 ± 0.011	0.837 ± 0.015	0.99	0.46	0.78	0.57
Light food intake (kcal/h)	0.067 ± 0.032	0.030 ± 0.015	0.63	0.075 ± 0.034	0.079 ± 0.033	0.99	0.59	0.35	0.50	0.049 ± 0.038	0.032 ± 0.011	0.93	0.103 ± 0.044	0.099 ± 0.039	1.00	0.76	0.10	0.85
Dark food intake (kcal/h)	0.088 ± 0.016	0.402 ± 0.130	0.0118	0.140 ± 0.032	0.407 ± 0.051	0.0327	0.0007	0.70	0.75	0.096 ± 0.027	0.330 ± 0.046	0.0061	0.122 ± 0.011	0.406 ± 0.082	0.0011	<0.0001	0.31	0.61
24 h food intake (kcal/h)	0.079 ± 0.015	0.174 ± 0.033	0.0338	0.110 ± 0.021	0.258 ± 0.030	0.0012	0.0001	0.0360	0.32	0.075 ± 0.011	0.196 ± 0.029	0.0215	0.113 ± 0.018	0.269 ± 0.050	0.0036	0.0002	0.09	0.58

Continues on next page.

Table S1: Summary of measured parameters, continued

	Males									Females								
	WT group	WT single	WT *P* value*	Brs3-/y group	Brs3-/y single	Brs3-/y *P* value	2-way ANOVA *P* value housing	genotype	interaction	Brs3+/- group	Brs3+/- single	Brs3+/- *P* value	Brs3-/- group	Brs3-/- single	Brs3-/- *P* value	2-way ANOVA *P* value housing	genotype	interaction
**35 °C Ambient temperature (Figure 5, continued)**																		
Light water intake (ml/h)	0.095 ± 0.008	0.334 ± 0.030	<0.0001	0.082 ± 0.005	0.299 ± 0.026	<0.0001	<0.0001	0.25	0.60	0.081 ± 0.027	0.317 ± 0.053	0.0084	0.066 ± 0.020	0.324 ± 0.082	0.0040	0.0001	0.94	0.83
Dark water intake (ml/h)	0.099 ± 0.025	0.135 ± 0.014	0.29	0.068 ± 0.011	0.150 ± 0.016	0.0067	0.0029	0.64	0.20	0.084 ± 0.019	0.204 ± 0.032	0.0122	0.098 ± 0.014	0.235 ± 0.039	0.0045	0.0002	0.43	0.76
24 h water intake (ml/h)	0.097 ± 0.015	0.216 ± 0.012	<0.0001	0.074 ± 0.007	0.198 ± 0.015	<0.0001	<0.0001	0.13	0.83	0.083 ± 0.006	0.244 ± 0.012	<0.0001	0.083 ± 0.009	0.200 ± 0.022	<0.0001	<0.0001	0.12	0.11
Light PA (counts/min)	57 ± 4	55 ± 6	0.95	56 ± 3	60 ± 5	0.85	0.88	0.74	0.57	27 ± 3	29 ± 3	0.94	29 ± 4	34 ± 5	0.66	0.43	0.37	0.71
Dark PA (counts/min)	52 ± 3	65 ± 9	0.34	66 ± 2	72 ± 9	0.80	0.18	0.14	0.60	45 ± 3	53 ± 6	0.38	54 ± 3	68 ± 5	0.07	0.0200	0.0180	0.49
24 h PA (counts/min)	54 ± 3	60 ± 8	0.68	61 ± 2	66 ± 6	0.78	0.32	0.24	0.91	37 ± 2	42 ± 5	0.47	43 ± 3	52 ± 3	0.09	0.0308	0.0248	0.48
**Fasting, CLAMS (Figure 6)**																		
Mean Tb (°C)	35.65 ± 0.11	35.27 ± 0.30	0.19	35.62 ± 0.06	35.24 ± 0.24	0.16	0.02	0.84	0.99	35.73 ± 0.09	35.47 ± 0.21	0.37	35.93 ± 0.08	35.14 ± 0.27	0.0007	0.0007	0.67	0.07
Minimum Tb (°C)	33.38 ± 0.16	32.38 ± 0.23	0.0037	33.05 ± 0.10	31.26 ± 0.37	<0.0001	<0.0001	0.0011	0.06	33.05 ± 0.16	30.77 ± 0.64	<0.0001	32.67 ± 0.15	30.41 ± 0.50	<0.0001	<0.0001	0.22	0.97
Tb span (°C)	3.46 ± 0.19	3.84 ± 0.42	0.45	3.91 ± 0.09	5.45 ± 0.27	<0.0001	0.0001	<0.0001	0.0136	4.03 ± 0.15	6.32 ± 0.66	<0.0001	4.43 ± 0.13	6.80 ± 0.46	<0.0001	<0.0001	0.13	0.88
Tb < 34 °C (% of time)	7.8 ± 1.6	17.9 ± 3.5	0.0118	10.6 ± 1.4	21.7 ± 3.8	0.0027	<0.0001	0.17	0.82	11.6 ± 1.8	19.7 ± 3.2	0.05	11.5 ± 1.5	25.1 ± 3.8	0.0007	<0.0001	0.30	0.27
Torpor episodes (number)	1.9 ± 0.3	4.6 ± 0.7	0.0005	2.9 ± 0.2	4.5 ± 0.7	0.0366	<0.0001	0.31	0.22	2.7 ± 0.3	3.0 ± 0.5	0.88	2.9 ± 0.3	2.7 ± 0.6	0.92	0.95	0.85	0.57
Torpor episode duration (min)	53.5 ± 9.0	49.8 ± 7.2	0.87	47.4 ± 5.9	57.3 ± 4.9	0.70	0.87	0.92	0.39	44.5 ± 5.9	70.1 ± 13.2	0.25	51.6 ± 8.5	108.3 ± 24.3	0.0032	0.0012	0.06	0.20
Longest torpor episode (min)	83.4 ± 0.0	102.1 ± 0.0	0.70	73.2 ± 0.0	126.6 ± 0.0	0.05	0.0397	0.68	0.31	77.1 ± 11.6	132.0 ± 38.2	0.06	76.6 ± 9.8	145.8 ± 13.1	0.0143	0.0009	0.70	0.68
Conductance (kcal/h/Δ°C)	0.025 ± 0.001	0.028 ± 0.002	0.18	0.026 ± 0.001	0.031 ± 0.002	0.0123	0.0025	0.17	0.48	0.024 ± 0.000	0.031 ± 0.002	<0.0001	0.025 ± 0.000	0.030 ± 0.000	<0.0001	<0.0001	0.28	0.06
Mean TEE (kcal/h)	0.308 ± 0.010	0.353 ± 0.017	0.22	0.331 ± 0.015	0.374 ± 0.030	0.25	0.0355	0.27	0.95	0.286 ± 0.006	0.370 ± 0.020	0.0003	0.315 ± 0.006	0.365 ± 0.012	0.0217	<0.0001	0.35	0.19
Minimum TEE (kcal/h)	0.145 ± 0.008	0.125 ± 0.007	0.30	0.147 ± 0.006	0.125 ± 0.016	0.24	0.0438	0.93	0.92	0.121 ± 0.005	0.113 ± 0.009	0.74	0.117 ± 0.007	0.111 ± 0.010	0.85	0.40	0.72	0.89
Mean RER	0.741 ± 0.006	0.701 ± 0.010	0.0008	0.739 ± 0.003	0.694 ± 0.006	0.0002	<0.0001	0.53	0.68	0.744 ± 0.005	0.729 ± 0.012	0.39	0.751 ± 0.005	0.728 ± 0.009	0.12	0.0319	0.76	0.62
Mean PA (counts/min)	76 ± 9	156 ± 13	0.0025	69 ± 4	169 ± 25	0.0003	<0.0001	0.87	0.50	77 ± 7	178 ± 33	0.0021	94 ± 5	176 ± 15	0.0114	<0.0001	0.70	0.61
24 h water intake (ml/h)	0.057 ± 0.010	0.087 ± 0.012	0.25	0.051 ± 0.014	0.092 ± 0.018	0.10	0.0190	0.99	0.72	0.066 ± 0.012	0.102 ± 0.021	0.37	0.096 ± 0.024	0.139 ± 0.018	0.25	0.06	0.10	0.86
**Fasting, telemetry (section 3.5)**																		
Mean Tb (°C)	35.52 ± 0.12	35.29 ± 0.32	0.62	35.52 ± 0.09	34.76 ± 0.29	0.0057	0.0076	0.14	0.14	35.11 ± 0.15	35.60 ± 0.14	0.08	35.45 ± 0.10	35.09 ± 0.13	0.25	0.69	0.59	0.0137
Minimum Tb (°C)	32.39 ± 0.16	32.57 ± 0.53	0.89	32.45 ± 0.11	30.03 ± 0.61	<0.0001	0.0003	<0.0001	<0.0001	30.31 ± 0.40	29.84 ± 0.52	0.71	31.40 ± 0.20	29.25 ± 0.56	0.0026	0.0049	0.57	0.06
Tb span (°C)	4.59 ± 0.20	3.53 ± 0.41	0.05	4.94 ± 0.13	6.47 ± 0.72	0.0021	0.48	<0.0001	0.0002	6.61 ± 0.43	6.74 ± 0.50	0.97	5.48 ± 0.17	7.84 ± 0.56	0.0014	0.0092	0.98	0.0188
Tb < 34 °C (% of time)	15.9 ± 1.4	14.3 ± 4.4	0.89	19.3 ± 1.9	31.9 ± 3.7	0.0018	0.0395	0.0002	0.0085	24.2 ± 2.1	19.6 ± 2.7	0.35	21.6 ± 1.6	23.1 ± 0.9	0.89	0.53	0.84	0.22
Torpor episodes (number)	3.9 ± 0.4	3.0 ± 0.5	0.39	3.4 ± 0.2	5.2 ± 0.7	0.0217	0.37	0.09	0.0092	3.1 ± 0.2	2.8 ± 0.5	0.88	3.7 ± 0.4	3.7 ± 0.6	0.99	0.70	0.11	0.80
Torpor episode duration (min)	57.7 ± 5.0	54.7 ± 12.9	0.98	81.1 ± 10.1	84.8 ± 13.7	0.97	0.99	0.0241	0.77	97.9 ± 7.5	89.1 ± 16.4	0.8111	84.6 ± 6.3	85.9 ± 16.3	0.99	0.73	0.44	0.64
Longest torpor episode (min)	113.8 ± 7.3	78.4 ± 18.9	0.63	144.9 ± 13.0	245.9 ± 81.4	0.0232	0.25	0.0010	0.0194	164.6 ± 17.6	129.6 ± 10.2	0.36	147.1 ± 10.9	154.1 ± 18.6	0.96	0.47	0.86	0.28

*adjusted P value from post hoc Šidák's test for group vs single housing.

Abbreviations:

Tb, core body temperature

TEE, total energy expenditure

RER, respiratory exchange ratio

PA, physical activity

Table S2. Effect of a range of ambient temperatures on thermal physiology of C57BL/6J mice

	**Males, single housed**	**Females, single housed**	**Males, group housed**	**Females, group housed**
**n of cages**	6	6	6	6
**n of data points in regression**	3654	4832		
**Body weight (g)**	28.0 ± 0.5	23.6 ± 0.3	29.8 ± 0.5	21.8 ± 0.3
**TEE analysis:**				
**TEE Slope, <Tlc_{EE} (kcal/h/°C)**	-0.0291 ± 0.0004	-0.0218 ± 0.0004		
**Tlc_{EE} (TNP_L, °C)**	28.45 ± 0.01	30.06 ± 0.19		
**TEE, at Tlc_{EE} (kcal/h)**	0.229 ± 0.006	0.204 ± 0.003		
**TEE_R (°C)**	35.52 ± 0.39	36.63 ± 0.28		
**TEE Slope, >TEE_R (kcal/h/°C)**	0.0350 ± 0.0240	0.0188 ± 0.0033		
**defended Tb (°C)**	36.31 ± 0.24	39.42 ± 0.36		
**Tb analysis:**				
**Tb, <Tb_{inc} (°C)**	35.59 ± 0.07	36.16 ± 0.13		
**Tb_{inc} (°C)**	30.39 ± 0.28	29.10 ± 0.20		
**Tb Slope, >Tb_{inc} <Tb_R (°C Tb/°C)**	0.191 ± 0.026	0.272 ± 0.015		
**Tb_R (°C)**	33.94 ± 0.08	34.65 ± 0.14		
**Tb Slope, >Tb_R (°C Tb/°C)**	0.967 ± 0.030	0.649 ± 0.014		
**Conductance analysis:**				
**n of data points in regression**	658	651	665	680
**Conductance slope, Ta<27°C (cal/h/°C/°C)**	0.013 ± 0.081	0.246 ± 0.063	0.838 ± 0.087	0.758 ± 0.076

Key to parameters:

TEE Slope, <Tlc_{EE} (kcal/h/°C)	TEE vs. Ta slope for Ta <Tlc_{EE}
Tlc_{EE} (TNP_L, °C)	lower critical temperature, the (first) breakpoint of the TEE vs. Ta graph; TNP_L
TEE, at Tlc_{EE} (kcal/h)	mean TEE at Ta = Tlc_{EE}
TEE_R (°C)	breakpoint of the TEE vs. Ta graph, where TEE starts to rise with Ta; TNP_D
TEE Slope, >TEE_R (kcal/h/°C)	TEE vs. Ta slope for Ta >TEE_R
defended Tb (°C)	defended Tb, X intercept of TEE vs. Ta line (using only Ta <Tlc_{EE})
Tb, <Tb_{inc} (°C)	mean Tb at Ta < Tb_{inc}
Tb_{inc} (°C)	Ta above which the Tb first increases; TNP_L
Tb Slope, >Tb_{inc} <Tb_R (°C Tb/°C)	Tb slope in the region >Tb_{inc} and <Tb_R
Tb_R (°C)	second breakpoint of Tb vs. Ta graph, where Tb starts to rise steeply; TNP_D
Tb Slope, >Tb_R (°C Tb/°C)	Tb vs. Ta slope for Ta >Tb_R
Conductance slope, Ta<27°C	Heat conductance vs. Ta slope for Ta<27°C

TEE and Tb parameters were determined by mixed model segmented linear regression [6]. The TEE model has three segments with the slope of the middle one fixed at zero. The Tb model has three segments with the slope of the first one fixed at zero. In both models, the two breakpoints and other two slopes are determined by the model. Conductance slope was obtained by linear regression. Data are means ± SE.

Table S3: Summary of adipose tissue weight, *UCP1* mRNA expression, and hormone levels

Males

	WT group	WT single	WT *P* value*	Brs3-/y group	Brs3-/y single	Brs3-/y *P* value*	2 way ANOVA housing	2 way ANOVA genotype	2 way ANOVA interaction
BAT weight (mg)	97.2 ± 4.8	81.9 ± 5.5	0.89	118.7 ± 6.5	114.2 ± 2.8	0.26	0.18	0.0005	0.46
iWAT weight (g)	0.289 ± 0.013	0.288 ± 0.022	0.62	0.334 ± 0.016	0.360 ± 0.032	1.00	0.56	0.0071	0.52
eWAT weight (g)	0.270 ± 0.012	0.267 ± 0.013	0.52	0.338 ± 0.015	0.311 ± 0.019	0.99	0.42	0.0031	0.52
BAT *Ucp1* mRNA	1.00 ± 0.19	1.75 ± 0.20	0.54	0.28 ± 0.04	0.49 ± 0.09	0.0035	0.0039	<0.0001	0.08
iWAT *Ucp1* mRNA	0.00245 ± 0.00116	0.00188 ± 0.00122	0.67	0.00064 ± 0.00034	0.00174 ± 0.00085	0.90	0.78	0.32	0.40
Serum T3 (ng/ml)	1.35 ± 0.06	1.54 ± 0.10	0.018	1.42 ± 0.07	1.11 ± 0.05	0.17	0.47	0.028	0.0030
Serum T4 (μg/dl)	4.67 ± 0.11	4.84 ± 0.14	0.920	4.64 ± 0.20	4.54 ± 0.18	0.79	0.87	0.388	0.4961
Serum leptin (ng/ml)	2.22 ± 0.20	1.19 ± 0.15	0.460	3.21 ± 0.29	2.70 ± 0.36	0.05	0.02	0.000	0.4238

Females

	Brs3+/- group	Brs3+/- single	Brs3+/- *P* value*	Brs3-/- group	Brs3-/- single	Brs3-/- *P* value*	2 way ANOVA housing	2 way ANOVA genotype	2 way ANOVA interaction
BAT weight (mg)	74.9 ± 3.4	88.1 ± 5.3	0.52	83.1 ± 5.5	91.9 ± 5.4	0.24	0.075	0.33	0.72
iWAT weight (g)	0.304 ± 0.015	0.316 ± 0.009	0.25	0.382 ± 0.038	0.302 ± 0.027	0.97	0.36	0.39	0.22
eWAT weight (g)	0.204 ± 0.009	0.212 ± 0.020	0.70	0.292 ± 0.039	0.253 ± 0.023	0.98	0.67	0.09	0.52
BAT *Ucp1* mRNA	0.65 ± 0.07	0.97 ± 0.07	0.37	0.77 ± 0.15	0.95 ± 0.06	0.058	0.018	0.62	0.47
iWAT *Ucp1* mRNA	0.00050 ± 0.00023	0.00228 ± 0.00151	0.43	0.00016 ± 0.00005	0.00016 ± 0.0001	0.40	0.10	0.71	0.97
Serum T3 (ng/ml)	0.80 ± 0.08	0.70 ± 0.09	0.085	0.96 ± 0.08	0.42 ± 0.08	0.98	0.11	0.91	0.19
Serum T4 (μg/dl)	4.67 ± 0.23	4.57 ± 0.32	0.001	5.19 ± 0.30	4.13 ± 0.55	0.71	0.00	0.55	0.03
Serum leptin (ng/ml)	2.58 ± 0.35	1.19 ± 0.15	0.756	3.87 ± 0.61	3.26 ± 0.40	0.98	0.54	0.10	0.74

* P value from post hoc Šidák multiple comparison test for group vs single housing
